# Immunotherapy targeting drug-tolerant *Mycobacterium tuberculosis* persisters accelerates tuberculosis cure in preclinical models

**DOI:** 10.1172/JCI196648

**Published:** 2026-02-03

**Authors:** Styliani Karanika, Tianyin Wang, Addis Yilma, Jennie Ruelas Castillo, James T. Gordy, Hannah Bailey, Darla Quijada, Kaitlyn Fessler, Rokeya Tasneen, Elisa M. Rouse Salcido, Farah Shamma, Harley T. Harris, Fengyixin Chen, Rowan E. Bates, Heemee Ton, Jacob Meza, Yangchen Li, Alannah D. Taylor, Jean J. Zheng, Jiaqi Zhang, Theodoros Karantanos, Amanda R. Maxwell, Eric Nuermberger, J David Peske, Richard B. Markham, Petros C. Karakousis

**Affiliations:** 1 Center for Tuberculosis Research, Division of Infectious Diseases, Department of Medicine, Johns Hopkins University School of Medicine, Baltimore, Maryland, USA.; 2 W. Harry Feinstone Department of Molecular Microbiology and Immunology, Johns Hopkins Bloomberg School of Public Health, Baltimore, Maryland, USA.; 3 Division of Hematological Malignancies, Department of Oncology, Sidney Kimmel Comprehensive Cancer Center, Johns Hopkins University Hospital, Baltimore, Maryland, USA.; 4 Department of Molecular and Comparative Pathobiology, Research Animal Resources, Johns Hopkins University, Baltimore, Maryland, USA.; 5 Department of Pathology, Sidney Kimmel Comprehensive Cancer Center, Johns Hopkins University Hospital, Baltimore, Maryland, USA.

**Keywords:** Immunology, Infectious disease, Therapeutics, Tuberculosis, Vaccines

## Abstract

*Mycobacterium tuberculosis* remains a global health crisis, ranking among the deadliest infectious diseases worldwide. In response to the WHO’s call for therapeutic vaccines to complement antibiotic regimens and reduce tuberculosis (TB) treatment duration, we developed an intranasal DNA vaccine fusing the *M. tuberculosis* stringent response gene *relMtb* with the gene encoding the DC-targeting chemokine *Mip3a* (also known as *CCL20*). Administered alongside the first-line regimen, this vaccine accelerated a stable cure in immunocompetent murine TB models, reducing lung inflammation and eliciting robust and sustained RelMtb-stimulated T cell responses systemically and locally. The *Mip3a/relMtb* vaccine enhanced DC recruitment, activation, and spatial coordination with T cells, suggesting improved innate-adaptive immune synergy. Notably, it augmented the efficacy of a novel drug-resistant TB regimen as well. Critically, the vaccine induced analogous antigen-stimulated T cell immunity in nonhuman primates, the gold standard for preclinical TB vaccine evaluation, with responses detected in blood and bronchoalveolar lavage mirroring those observed in the murine models. These findings underscore the potential of this strategy to advance therapeutic TB vaccine development targeting *M. tuberculosis* persisters while providing a framework to define correlates of vaccine-mediated protection.

## Abstract 2

An intranasal DNA vaccine accelerated tuberculosis cure and achieved better outcomes than standard or drug-resistant regimens alone.

## Graphical abstract

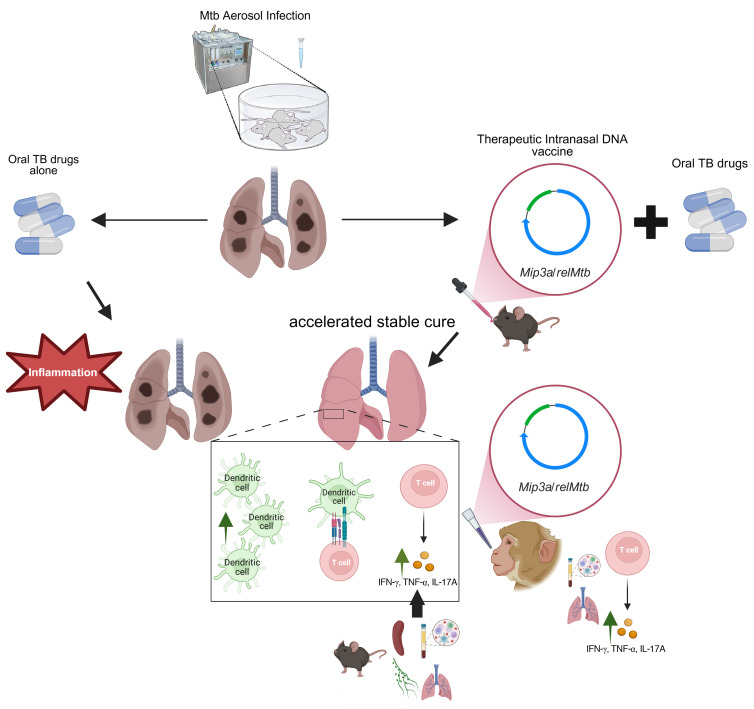

## Introduction

In 2024, *Mycobacterium tuberculosis* surpassed SARS-CoV-2 to again become the leading global cause of death due to a single infectious agent (1). Approximately 8.2 million people were newly diagnosed with tuberculosis (TB) in 2023 — the highest number reported since the WHO began global TB monitoring in 1995 (1). The current standard 6-month regimen, comprising rifampin (R), isoniazid (H), pyrazinamide (Z), and ethambutol (E), is highly effective against drug-susceptible TB. However, its prolonged duration and complexity often lead to treatment interruptions that jeopardize cure rates and promote the emergence of drug-resistant (DR) TB (2, 3), which remains a public health crisis (4). Although newer TB drugs, such as bedaquiline (B), pretomanid (Pa), and linezolid (L), offer hope for improved treatment of individuals with DR TB, resistance to each of these agents has already emerged, raising concerns about the long-term effectiveness of current antibiotic regimens for DR TB (5–7).

Given all of the above, the WHO has strongly advocated for the development of therapeutic TB vaccines as a strategy to address current limitations in TB treatment, setting as one of the priorities the shortening of drug treatment without subsequent relapse in patients with active TB disease (8). A small number of therapeutic TB vaccines, including RUTI (9), *M*. *indicus pranii* (10), *M*. *vaccae* (11), ID93+GLA-SE (12), and *Mip3a/relMtb* (13), have been evaluated in preclinical models and demonstrated varying degrees of adjunctive efficacy when combined with TB drugs. However, to date, none of these vaccines has been shown to reduce the duration of curative TB therapy without subsequent relapse following treatment completion. Interestingly, while the H56:IC31 vaccine demonstrated preclinical efficacy in preventive and post-exposure settings (14, 15), it has not been evaluated as an adjunct to standard drug therapy in preclinical models and failed to prevent recurrence in a recent clinical trial (16).

We have previously shown that intranasal (IN) delivery of the therapeutic DNA vaccine (*Mip3a/relMtb*) containing a fusion of the *M. tuberculosis* stringent response gene *relMtb (Rv2583c)* with the gene encoding the immature DC–targeting chemokine *Mip3a/CCL20*, when combined with H, substantially decreased the lung bacillary burden compared with H alone (13). The rationale for selecting RelMtb as the therapeutic vaccine antigen was based on its central role in bacterial persistence during antibiotic therapy. RelMtb mediates the stringent response, enabling *M. tuberculosis* to enter metabolic dormancy under stress conditions including antibiotic exposure, nutrient starvation, and hypoxia (17, 18). This adaptive state, which is characterized by reduced replication and (p)ppGpp accumulation, directly underlies *M. tuberculosis* antibiotic tolerance and bacterial persistence, the primary obstacles to treatment shortening. Critically, *M. tuberculosis* strains lacking functional RelMtb exhibit drastically reduced persistence during chronic infection and impaired survival under stress conditions, while inhibiting RelMtb blocks Mtb entry into quiescence and enhances H potency (17, 19). Moreover, H treatment has been shown to differentially shape the antigenic environment in infected lungs: RelMtb-reactive CD4^+^ T cells significantly increase following treatment. However, T cell responses to the early secreted antigenic target of 6 kDa protein (ESAT6), an *M. tuberculosis* secretory protein and immunodominant and potent T cell antigen, decrease as actively replicating bacteria are eliminated (3). This differential antigen availability makes RelMtb a promising therapeutic target for eliminating the *M. tuberculosis* persister subpopulation responsible for relapse.

Expanding on our previous findings, we now show that adjunctive use of the vaccine with the standard rifampin-isoniazid-phrazinamide-ethambutol (RHZE) regimen accelerated lung culture conversion and prevented post-treatment relapse in immunocompetent mice. The vaccine also enhanced the efficacy of the drug-resistant TB regimen involving bedaquiline-pretomanid-linezolid (BPaL), further reducing the lung bacterial burden. In a second murine model more closely representing human TB pathology, the vaccine decreased lung inflammation while improving RHZE-driven bacterial clearance. Mechanistically, IN vaccination boosted DC recruitment and activation, correlating with elevated antigen-stimulated T cell production of cytokines critical for TB control. Parallel studies in nonhuman primates revealed analogous induction of protective immune responses, indicating the translational potential of this strategy to shorten TB treatment durations across diverse clinical settings.

## Results

### IN immunization with the Mip3a/relMtb fusion enhances the efficacy of the first-line regimen for drug-susceptible TB in immunocompetent C57BL6 mice.

**Figure 1:**
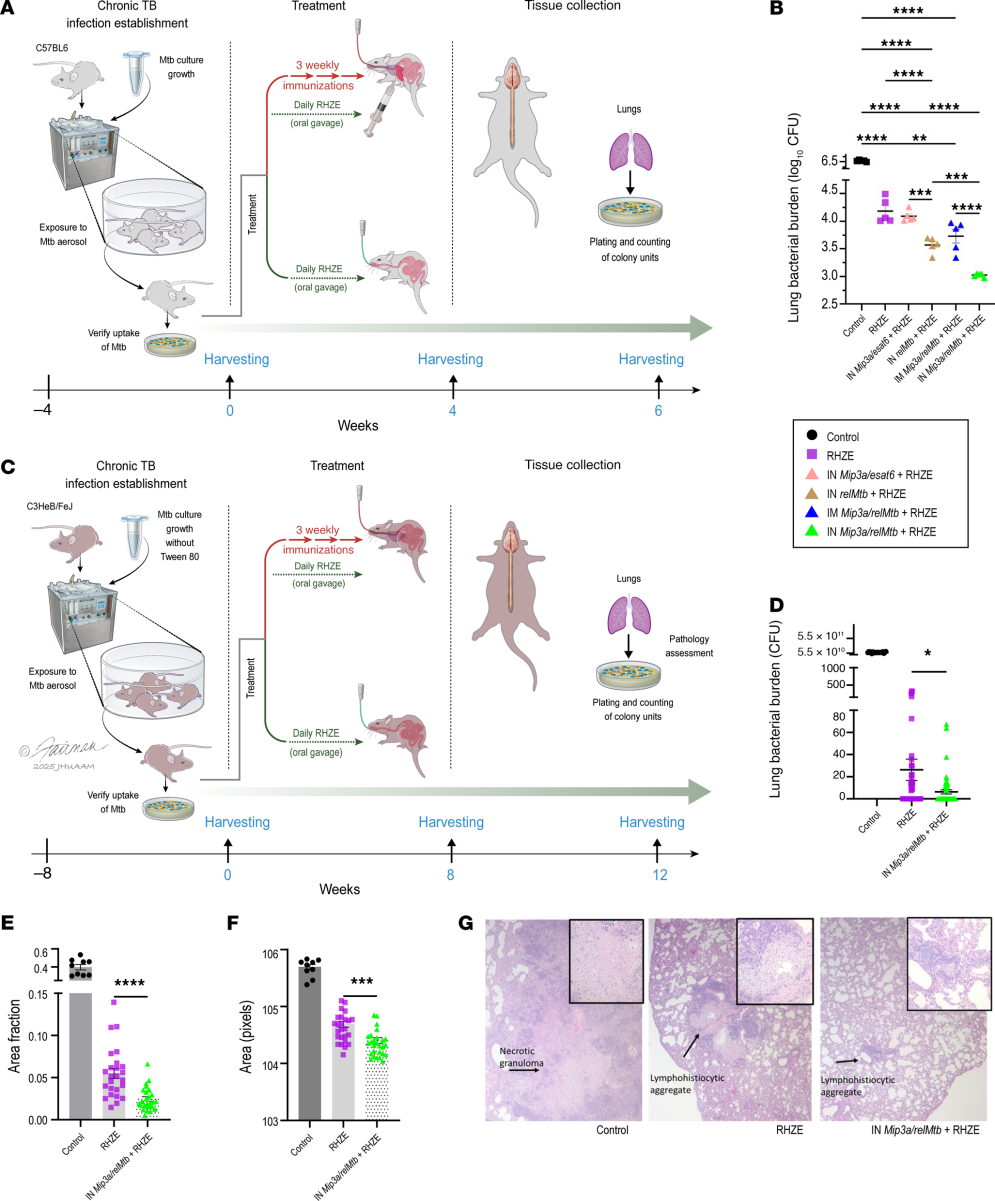
Therapeutic IN administration of the *Mip3a/relMtb* fusion vaccine enhances the efficacy of the first-line regimen for drug-susceptible TB and reduces lung inflammation in immunocompetent mice. (**A**) Experimental timeline of *M. tuberculosis* challenge study of C57BL/6 mice. Illustration credit: Jennifer Fairman. (**B**) Scatterplot of lung mycobacterial burden at 6 weeks in C57BL/6 mice (*n* = 5 mice/group); (**C**) Experimental timeline of *M. tuberculosis* challenge of C3HeB/FeJ mice. Illustration credit: Jennifer Fairman. (**D**) Scatterplot of lung mycobacterial burden at 12 weeks in C3HeB/FeJ mice (*n* = 19 for the control group, *n* = 55 for the RHZE group, *n* = 55 for the IN *Mip3a/relMtb* plus RHZE group). (**E**) Lymphohistiocytic aggregates (with necrosis or no necrosis) divided by the total lung area per slide in C3HeB/FeJ mice. (**F**) average area of inflamed foci per C3HeB/FeJ mouse (*n* = 10 for control group, *n* = 25 for the RHZE group, *n* = 27 for IN *Mip3a/relMtb* plus RHZE), assessment was performed on sections encompassing the entire left lungs, collected 12 weeks post treatment initiation and 20 weeks post infection to promote granuloma formation; (**G**) widespread granulomatous inflammation, including large central necrotic areas were observed in the lung tissues of control C3HeB/FeJ mice in contrast to the lungs receiving either RHZE or IN *Mip3a/relMtb* fusion vaccine plus RHZE which showed foci of reduced inflammation, mostly consistent with scattered lymphocytic infiltrates surrounding foamy histiocytes, but no areas of necrosis. Representative images for each C3HeB/FeJ group are shown (original magnification, ×40 and ×200 [inserts]). *P < 0.05 **P < 0.01, ***P < 0.001, and ****P < 0.0001, by 1-way ANOVA followed by Tukey’s multiple-comparison test (**B**) or unpaired 2-tailed *t* test (**D**–**F**). Data are shown as the mean ± SEM.

In the current study, we aimed to test the therapeutic efficacy of IN administration of the *Mip3a/relMtb* fusion vaccine in combination with the complete first-line regimen for drug-susceptible TB (RHZE) in C57BL/6 mice. Beginning 4 weeks after *M. tuberculosis* aerosol infection, mice were treated daily with human-equivalent doses of oral RHZE for a total of 6 weeks (Figure 1A). Separate groups of mice received either the *Mip3a/relMtb* fusion vaccine or the original vaccine expressing only the antigen (*relMtb*), each of which was administered once weekly via the IN or intramuscular (IM) route for 3 weeks. The control groups were vaccinated with vehicle and received mock gavage (control) or the first-line regimen (RHZE). An additional control group received a fusion vaccine containing the *Mip3a* gene fused to the gene encoding ESAT6 (*Mip3a/esat6)*, rather than RelMtb, a stringent response antigen (20–22).

Six weeks after primary vaccination, the most substantial reduction in the mean lung bacillary burden was observed in the group receiving the IN *Mip3a/relMtb* fusion vaccine along with RHZE compared with any other vaccination approach, RHZE alone, or control (absolute reduction of mycobacterial burden: >0.5, 1.3 and 3.6 log_10_ CFU; *P* < 0.001, *P* < 0.0001 and *P* < 0.0001, respectively; Figure 1B and Supplemental Figure 1; supplemental material available online with this article; https://doi.org/10.1172/JCI196648DS1). Interestingly, we observed no adjunctive therapeutic activity after IN vaccination with *Mip3a/esat6* compared with RHZE alone (Figure 1B). Thus, collectively, the IN *Mip3a/relMtb* fusion vaccine was found to be the most effective vaccination strategy, resulting in the most significant mycobacterial reduction in the lungs when combined with the first-line treatment for drug-susceptible TB in C57BL/6 mice.

To further characterize the immunological differences underlying this differential therapeutic efficacy, we measured secreted cytokines in bronchoalveolar lavage (BAL) supernatants 6 weeks after prime vaccination using Luminex multiplex cytokine analysis. Mice receiving the IN *Mip3a/relMtb* vaccine exhibited significantly elevated TNF-α (*P* = 0.0407) and IL-17A (*P* = 0.0360) secretion compared with IN *Mip3a/Esat6*-vaccinated mice, further supporting the idea of antigen-dependent immune responses rather than general immune activation and aligning with the superior therapeutic efficacy of the *Mip3a/relMtb* vaccine (Supplemental Figure 2). Secreted IFN-γ levels were comparable between groups (*P* = 0.4965), likely reflecting contributions from multiple IFN-γ–producing populations including NK cells, innate lymphoid cells, and γδ T cells, which probably respond to nonspecific inflammatory signals (Supplemental Figure 2).

Multiplex immunofluorescence analysis of lung tissue sections from the same cohort revealed that *Mip3a/relMtb* vaccination promoted significantly enhanced DC-T cell spatial organization compared with *Mip3a/Esat6*. Specifically, we found that significantly more DCs were localized within 5 μm of T cells in *Mip3a/relMtb*-vaccinated mice (median 7 DCs per T cell) compared with *Mip3a/Esat6*-vaccinated mice (median 0 DCs per T cell, *P* = 0.0159), suggesting enhanced DC–T cell interactions that may contribute to the superior therapeutic efficacy of the RelMtb-targeted vaccine (Supplemental Figure 3). Our analysis also revealed a trend toward increased DC infiltration into the lungs of mice receiving IN *Mip3a/relMtb* (median 0.7% of total cells) compared with IN *Mip3a/esat6* (median 0.2%), although this did not reach statistical significance (*P* = 0.096), probably due to the high inter-animal variability and small sample size (Supplemental Figure 3).

### IN immunization with the Mip3a/relMtb fusion enhances the efficacy of the first-line regimen for drug-susceptible TB in immunocompetent C3HeB/FeJ mice.

Next, we investigated the efficacy of the IN *Mip3a/relMtb* fusion vaccine in a second mouse model, C3HeB/FeJ mice, which develop necrotic lung granulomas following aerosol infection with *M. tuberculosis* (23). In this experiment, to further augment the virulence of the mycobacterial culture and promote robust granuloma development, we implemented a chronic infection model without including Tween-80 (24) and extended the waiting period from 4 to 8 weeks after aerosol infection, prior to treatment initiation (Figure 1C). Mice were treated daily with human-equivalent doses of oral RHZE for 12 weeks. Twelve weeks after primary vaccination, we observed a significant reduction in the mean lung bacillary burden in the group receiving the IN *Mip3a/relMt* fusion vaccine along with RHZE compared with those that received RHZE alone (6 vs. 26 CFU; *P* = 0.0468, Figure 1D). Of note, the normalized lung weights (lung-to-body weight ratios), an established surrogate of gross lung inflammation (25), were more profoundly decreased in the group receiving the IN *Mip3a/relMtb* fusion vaccine plus RHZE compared with RHZE alone (*P* = 0.0101; Supplemental Figure 4).

Histologically, widespread granulomatous inflammation, including large central necrotic areas containing very high loads of acid-fast bacilli (AFB), were observed in the lung tissues of control C3HeB/FeJ mice (Supplemental Figure 5). In contrast, the lungs of C3HeB/FeJ mice receiving either RHZE or RHZE plus *Mip3a/relMtb* fusion vaccine showed foci with reduced inflammation, mostly consistent with scattered lymphocytic infiltrates surrounding foamy histiocytes, but no areas of necrosis. Both treatment groups had only rare AFB in the lesions, which could not be reliably quantified through pathology (Supplemental Figure 5). Quantitatively, the group receiving the IN *Mip3a/relMtb* fusion vaccine plus RHZE exhibited a significant reduction in the surface area involved by lymphohistiocytic aggregates expressed as a percentage of the total lung area per slide and a decreased mean area of inflammatory foci per animal compared with the RHZE alone group (*P* < 0.0001, Figure 1E; *P* < 0.0006, Figure 1F). Representative images are shown in Figure 1G. Thus, collectively, the IN *Mip3a/relMtb* fusion vaccine enhanced the bactericidal activity of RHZE and reduced TB-induced lung inflammation in a second mouse model with human-like TB pathology.

### Adjunctive IN Mip3a/relMtb fusion vaccine shortens the duration of curative TB treatment in a relapse model.

**Figure 2:**
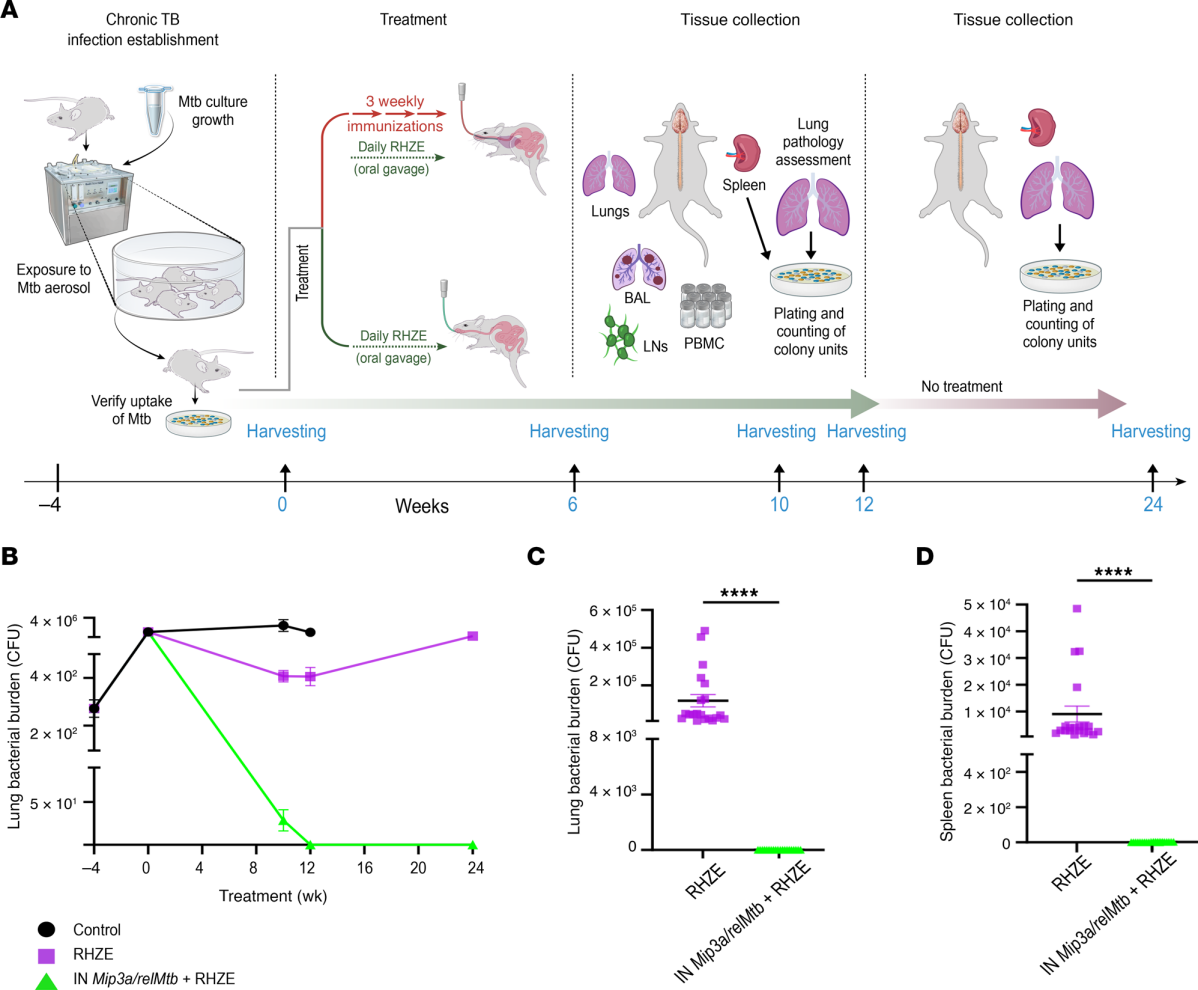
Adjunctive IN *Mip3a/relMtb* fusion vaccine leads to lung-culture negativity more rapidly than RHZE alone, yielding a stable cure after 12 weeks of treatment in immunocompetent C57BL6 mice. (**A**) Timeline of the *M. tuberculosis* challenge study. Illustration credit: Jennifer Fairman. (**B**) Timeline of the mean lung mycobacterial burden 10, 12, and 24 weeks after the primary vaccination, per vaccination group. Scatterplot of the lung (**C**) and splenic (**D**) mean mycobacterial burden 24 weeks after primary vaccination (and 12 weeks after treatment discontinuation) (*n* = 14–20/group). ****P < 0.0001, by Mann-Whitney *U* test.

Next, we evaluated whether the adjunctive IN *Mip3a/relMtb* fusion vaccine could reduce the time required to achieve sterilization of lung tissues. Beginning 4 weeks after *M. tuberculosis* aerosol infection, C57BL/6 mice were treated daily with human-equivalent doses of oral RHZE for a total of 12 weeks (Figure 2A). At 10 weeks after primary vaccination, mice receiving the IN *Mip3a/relMtb* fusion vaccine had a significant reduction in mean lung CFU compared with control mice treated with RHZE alone (additional reduction in mycobacterial burden: 379 CFU, *P* < 0.0001; Figure 2B). Twelve weeks after primary vaccination, the lungs of all mice receiving the IN *Mip3a/relMtb* fusion vaccine plus RHZE had negative cultures in contrast to those that received RHZE alone (0 CFU vs. 406 CFU, *P* < 0.0001; Figure 2B). Upon completion of 12 weeks of treatment, RHZE was discontinued and mice were held for an additional 12 weeks to assess microbiological relapse in the lungs and spleen. As expected, all 20 mice in the RHZE group had positive lung cultures, since they never achieved culture negativity at the end of treatment (Figure 2, C and D). In contrast, none of the mice (0 of 20) that had received the IN *Mip3a/relMtb* fusion vaccine experienced microbiological relapse (Figure 2, C and D). Thus, IN delivery of the *Mip3a/relMtb* fusion vaccine shortened the duration of curative treatment with the first-line regimen in *M. tuberculosis*–infected mice.

### IN immunization with the Mip3a/relMtb fusion vaccine induces durable TB-protective immune responses when combined with the first-line regimen for drug-susceptible TB.

**Figure 3:**
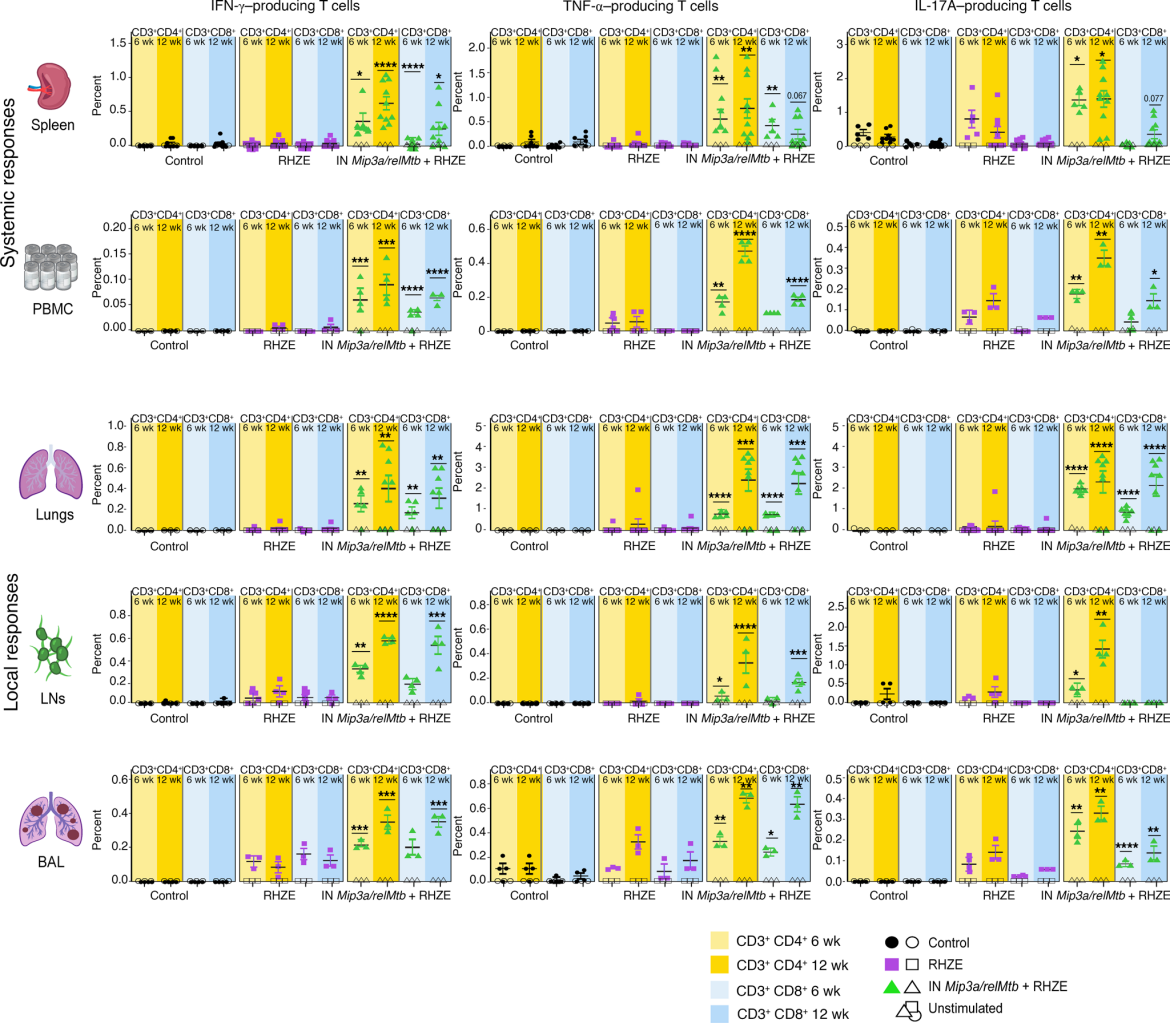
Therapeutic IN *Mip3a/relMtb* fusion immunization induces durable local and systemic TB-protective T cell immune responses when combined with the first-line regimen for drug-susceptible TB regimen (RHZE) in immunocompetent C57BL6 mice. Six and 12-week RelMtb-stimulated CD4^+^ and CD8^+^ T cells producing IFN-γ, TNF-α, or IL-17A following ex vivo stimulation with purified recombinant RelMtb protein in spleens and PBMCs (representing systemic responses) and lungs, LNs, and BAL (representing local responses). Filled symbols represent RelMtb-stimulated responses; open symbols represent unstimulated controls. Values shown are the percentages of cytokine-producing cells within the indicated T cell population. *n* = 5–10/group. To enhance clarity, we present statistically significant pairwise comparisons of RelMtb-stimulated T cell subpopulations at identical time points between the IN *Mip3a/relMtb* vaccine plus RHZE and RHZE alone. 6w, 6 weeks; 12w, 12 weeks. *P < 0.05, **P < 0.01, ***P < 0.001, and ****P < 0.0001, by 1-way ANOVA. Illustration credit: Jennifer Fairman.

**Figure 4:**
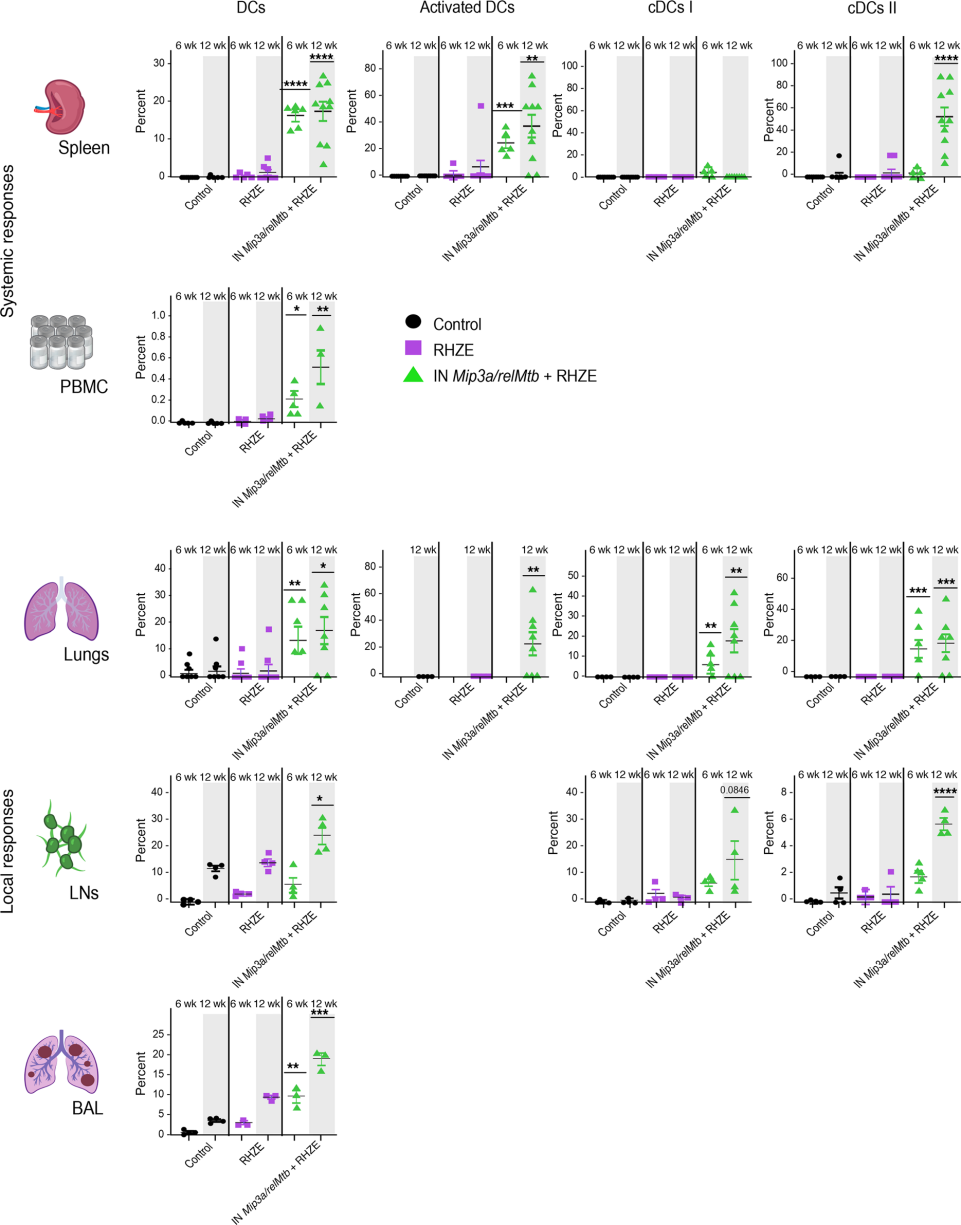
Therapeutic IN *Mip3a/relMtb* fusion immunization induces sustained DC recruitment and activation locally and systemically when combined with the first-line regimen for drug-susceptible TB regimen (RHZE) in immunocompetent C57BL6 mice. Six and 12-week percentage of DCs, activated DCs, cDCs I and II in spleen and PBMCs (representing systemic responses) and lungs, LNs, and BAL (representing local responses); *n* = 5–10/group. To enhance clarity, we present statistically significant comparisons of DC populations at identical time points between the IN *Mip3a/relMtb* vaccine plus RHZE and RHZE alone. DCs, total cDCs. *P < 0.05, **P < 0.01, ***P < 0.001, and ****P < 0.0001, by 1-way ANOVA followed by Tukey’s multiple-comparison test. Illustration credit: Jennifer Fairman.

We measured the immune responses across the different groups over time (6 and 12 weeks after treatment; Figure 3). Compared with the RHZE group, the RHZE plus IN *Mip3a/relMtb* fusion vaccine resulted in a significantly higher percentage of RelMtb-stimulated CD4^+^ and CD8^+^ T cells producing IFN-γ, TNF-α, and IL-17A, which are important cytokines for optimal TB control (26–28), both systemically (spleen and PBMCs) and at the site of infection (lungs, mediastinal lymph nodes [LNs], and BAL) (Figure 3). This vaccination strategy also resulted in increased percentages of total classical DCs (cDCs), activated DCs, and their major subtypes (cDC types I and II [cDC I and cDC II]) relative to RHZE alone, consistent with increased DC recruitment and activation, as well as enhanced antigen presentation and CD4^+^ and CD8^+^ T cell response priming (Figure 4). These responses were sustained at 12 weeks after treatment (Figures 3 and 4).

### Immunological profiles associated with mycobacterial control.

We used simple linear regression models to test whether IN fusion vaccine–induced peak antigen-responsive T cell–producing cytokines and/or DC proportions in relevant tissues (spleen, lungs, LNs, PBMCs, and BAL) were associated with lung bacillary burden. Higher percentages of RelMtb-stimulated IL-17A^+^, IFN-γ^+^, and TNF-α^+^ T cells in PBMCs and BAL, along with RelMtb-stimulated IFN-γ^+^ and TNF-α^+^ T cells in LNs and total cDCs in BAL were highly and significantly associated with lower lung bacillary burden (Supplemental Figure 6). These findings suggest that these vaccine-induced immune responses may serve as indirect surrogates of optimal TB control.

### IN immunization with the Mip3a/relMtb fusion vaccine promotes DC infiltration into the lungs and enhances DC colocalization with T cells.

**Figure 5:**
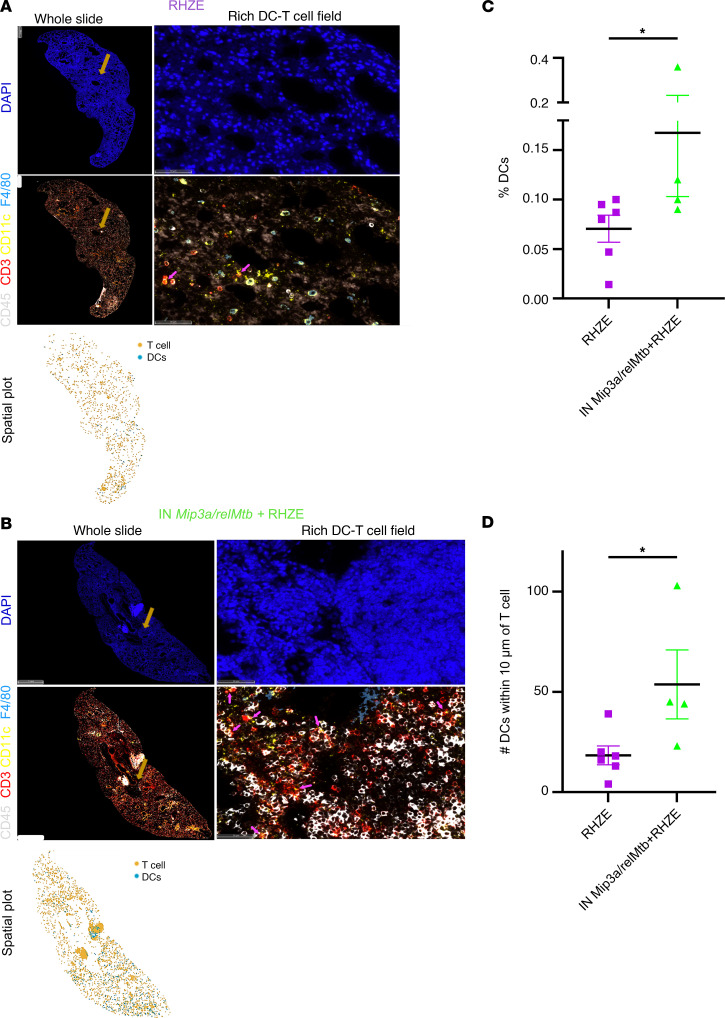
Therapeutic IN *Mip3a/relMtb* fusion immunization induces local DC infiltration, enhancing colocalization with T cells in mouse lungs. (**A** and **B**) Representative lung sections (whole slides, rich DC–T cell fields of view and DC–T cell spatial plots) from RHZE alone (**A**) versus IN *Mip3a/relMtb* plus RHZE after 12 weeks of treatment (**B**) stained with antibodies for DAPI^+^ only (dark blue, cell nuclei, top) or CD45^+^ (white, hematopoietic cells, bottom), CD45^+^CD3^+^ (red, T cells, bottom), and CD45^+^CD3^-^CD11c^+^F4/80^–^ (yellow, DCs, bottom). Light brown arrows in low-magnification images (scale bars: 1 mm) indicate the specific areas shown in the corresponding high-magnification images (scale bars: 50 μm). Magenta arrows indicate DC–T cell colocalization. (**C** and **D**) Quantification of total DCls per DAPI^+^ cells (percentage) and colocalization of DCs and T cells defined as the number of DCs within 10 μm of T cells (RHZE alone [*n* = 6] vs. IN *Mip3a/relMtb* plus RHZE [*n* = 4]). Assessment was performed on sections encompassing the entire left lung. *P < 0.05, by Mann-Whitney *U* test.

We used multiplex immunofluorescence to quantify DCs and T cells in murine lungs collected 12 weeks after RHZE with or without prime vaccination (Figure 5, A and B, and Supplemental Figure 7). Using microscopy, we also assessed DC and T cell colocalization as an indirect measure of potentially enhanced DC–T cell interactions (Figure 5, A and B). Mice receiving the fusion vaccine and RHZE had a significantly higher percentage of total DCs (defined as DCs/DAPI^+^ cells) in the lungs than did those receiving RHZE alone (*P* = 0.043, Figure 5C), suggesting a significantly greater local DC infiltration. The percentage of total (including non-antigen-stimulated) T cells did not differ significantly between the 2 groups (*P* = 0.352, Supplemental Figure 8). Next, we measured the number of DCs colocalized within 10 μm T cells, a distance equal to a cell diameter, as a surrogate marker for cell-to-cell interactions. We found that mice receiving the fusion vaccine plus RHZE had a significantly higher number of DCs within 10 μm T cells than did the group receiving RHZE alone (median number 44 DCs vs. 17 DCs, *P* = 0.019; Figure 5D). These findings suggest that the IN fusion vaccine strategy induced local DC infiltration and enhanced DC engagement of T cells.

### IN immunization with the Mip3a/relMtb fusion vaccine enhances the bactericidal activity of a DR-TB regimen in immunocompetent mice.

**Figure 6:**
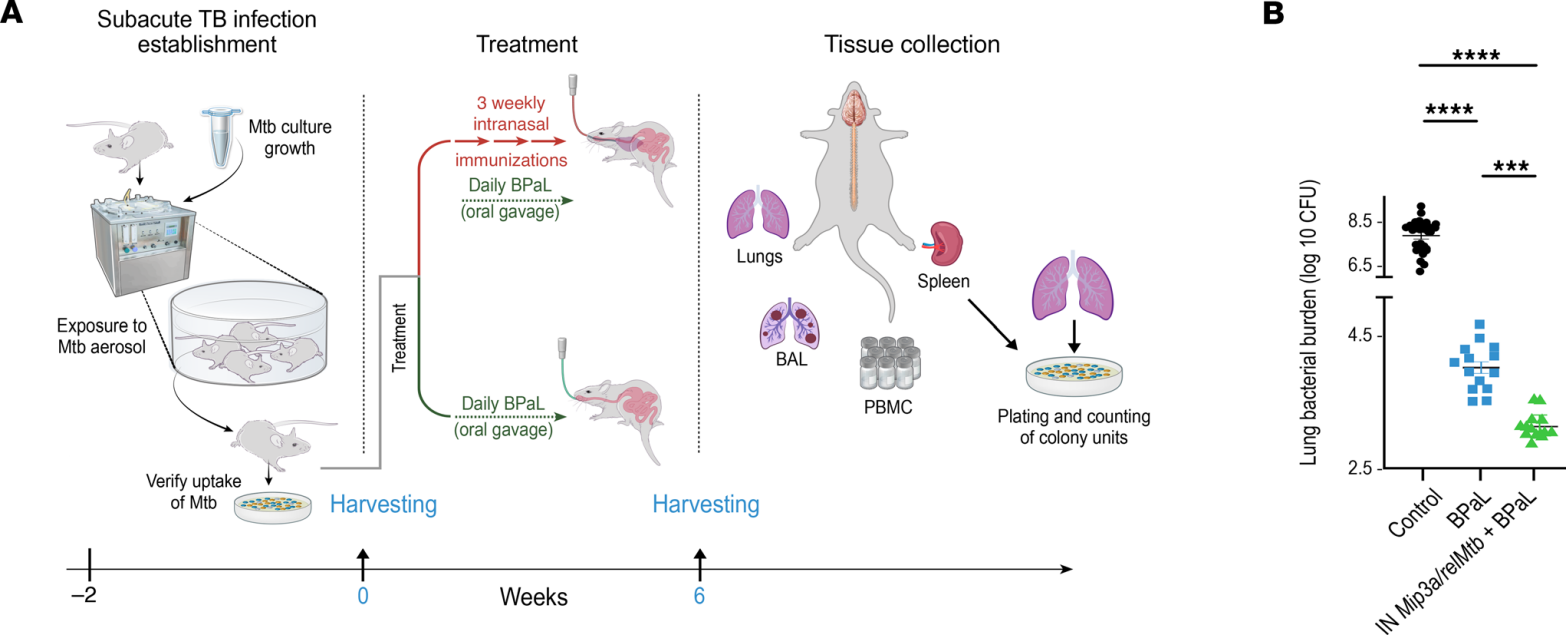
IN *Mip3a/relMtb* DNA fusion vaccine enhances the activity of the potent DR-TB regimen BPaL in immunocompetent C57BL6 mice. (**A**) Timeline of *M. tuberculosis* challenge study. Illustration credit: Jennifer Fairman. (**B**) Scatterplot of the mean lung mycobacterial burden 6 weeks after treatment initiation (*n* = 10–15/group). ***P < 0.001 and ****P < 0.0001, by 1-way ANOVA followed by Tukey’s multiple-comparison test.

Next, in order to determine whether the adjunctive therapeutic efficacy of the IN *Mip3a/relMtb* fusion vaccine is independent of the anti-tubercular regimen, we assessed its activity in combination with a novel oral regimen for DR-TB comprising BPaL (29, 30) compared with BPaL alone. For these studies, we selected the subacute model of TB infection (i.e., higher *M. tuberculosis* inoculum with a shorter incubation period before treatment initiation) instead of a chronic model to allow a sufficiently dynamic range in which to assess any additional mycobactericidal activity of the vaccine beyond that of the already potent BPaL regimen. Two weeks after high-dose *M. tuberculosis* aerosol infection, mice received daily oral treatment with human-equivalent doses of BPaL for 6 weeks (Figure 6A). Relative to those receiving BPaL alone, mice receiving the BPaL plus IN fusion vaccine had a significantly greater reduction in the lung mycobacterial load (CFU reduction difference: 0.7 log_10_, *P* < 0.001; Figure 6B and Supplemental Figure 9) and a nonsignificant reduction in the splenic mycobacterial burden (CFU reduction difference: 0.3 log_10_, *P* = 0.8, Supplemental Figure 10) at 6 weeks of treatment. These findings suggest that IN immunization with the fusion vaccine offered adjunctive therapeutic efficacy independently of the drug treatment regimen.

### IN immunization with the Mip3a/relMtb fusion vaccine induces TB-protective immune responses when combined with a potent DR-TB regimen.

**Figure 7:**
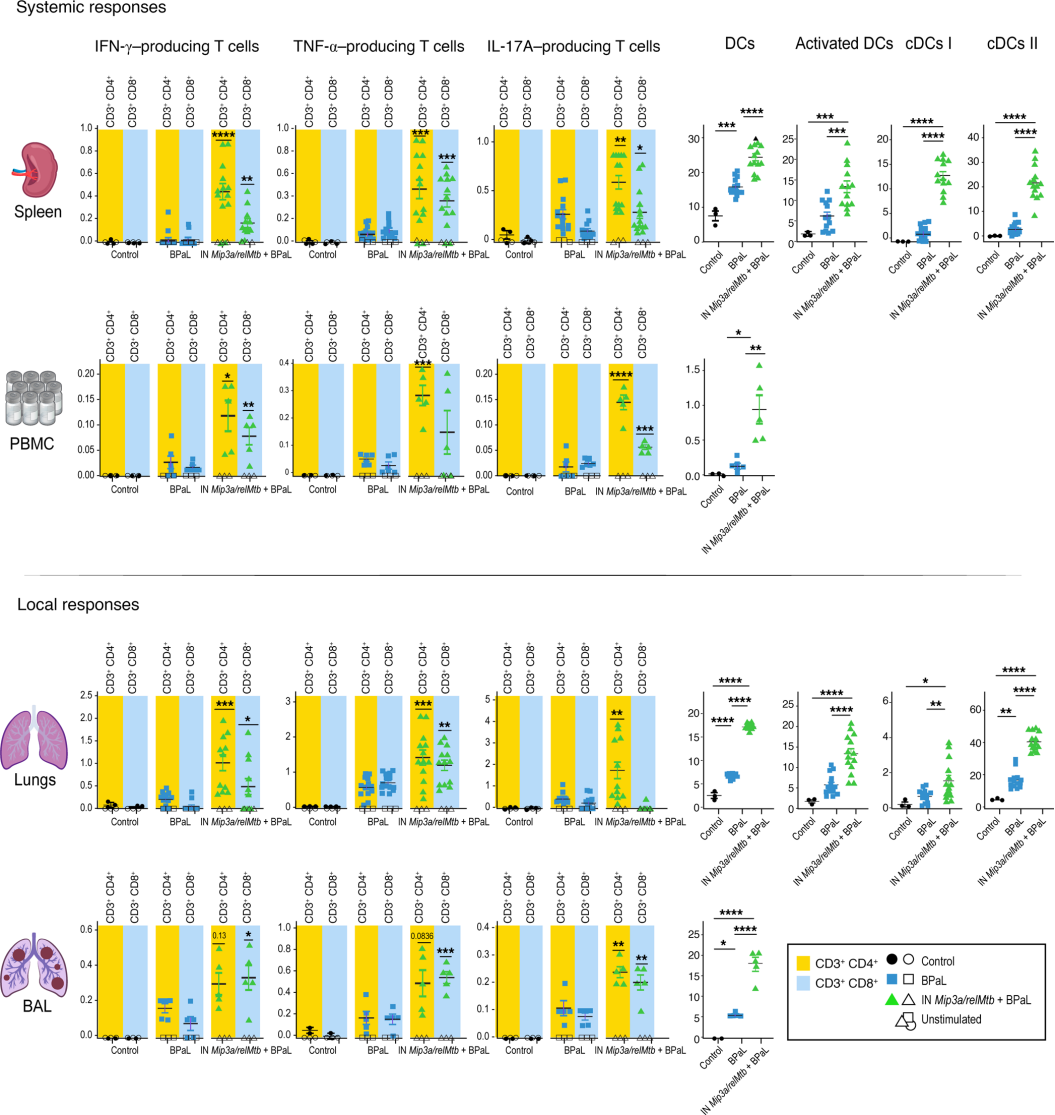
IN *Mip3a/relMtb* DNA fusion induces TB-protective immune responses when combined with the potent DR-TB regimen BPaL in immunocompetent mice. Six-week RelMtb-stimulated CD4^+^ and CD8^+^ T cells producing IFN-γ, TNF-α, or IL-17A following ex vivo stimulation with purified recombinant RelMtb protein and the percentage of DCs, activated DCs, and cDCs I and II in the spleen and PBMCs (representing systemic responses), lungs, and BAL (representing local responses). Filled symbols represent RelMtb-stimulated responses; open symbols represent unstimulated controls. Values shown are percentages of cytokine-producing cells within the indicated T cell population or DC frequencies within total cells. *n* = 10–15/group. To enhance clarity, statistically significant comparisons of RelMtb-stimulated T cell or DC subpopulations between IN *Mip3a/relMtb* vaccine plus BPaL and BPaL alone are shown. *P < 0.05, **P < 0.01, ***P < 0.001, and ****P < 0.0001, by 1-way ANOVA followed by Tukey’s multiple-comparison test. Illustration credit: Jennifer Fairman.

Next, we measured both local and systemic immune responses following 6 weeks of treatment with BPaL with or without IN fusion vaccination (Figure 7). Similar to the RHZE study, compared with the BPaL-only group, IN fusion vaccination combined with BPaL led to significantly higher percentages of RelMtb-stimulated CD4^+^ and CD8^+^ T cells producing IFN-γ, TNF-α, or IL-17A, both systemically (spleen and PBMCs) and at the site of infection (lungs and BAL) (Figure 7). The BPaL plus IN fusion vaccine group also had significantly higher percentages of total cDCs, activated DCs, and their major subtypes (cDC I and cDC II) relative to the BPaL-only group (Figure 7).

### The IN Mip3a/relMtb fusion vaccine is immunogenic in nonhuman primates.

**Figure 8:**
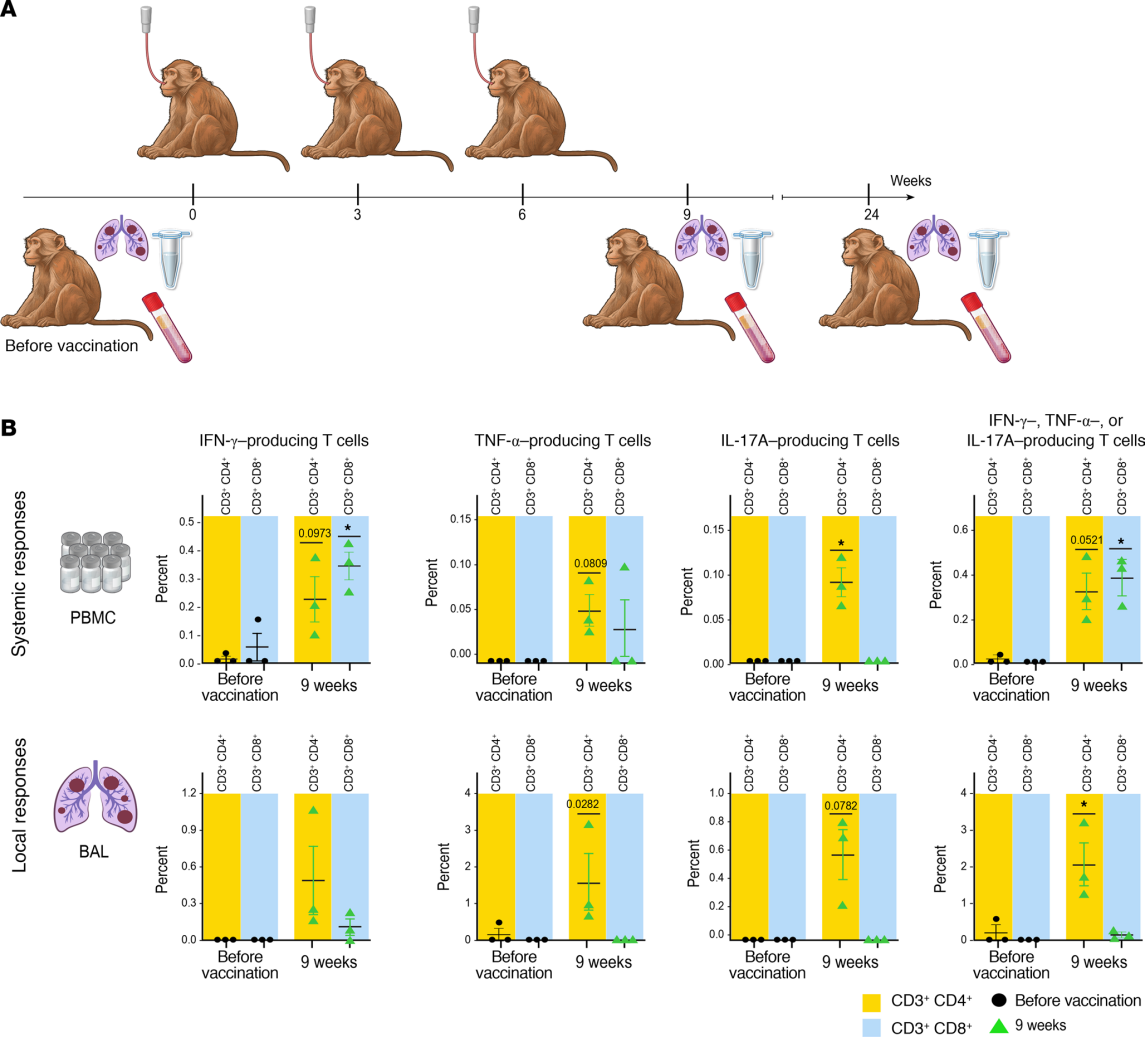
IN *Mip3a/relMtb* DNA fusion vaccine induces RelMtb-stimulated T cell responses in nonhuman primates consistent with protective immunity observed in mice. (**A**) Timeline of the immunogenicity study. (**B**) Nine-week RelMtb-stimulated CD4^+^ and CD8^+^ T cells producing IFN-γ, TNF-α, or IL-17A in PBMCs and BAL. T cell responses are expressed in percentages. *n* = 3. Direct comparisons of T cell subpopulation dynamics before and 9 weeks after prime vaccination are shown. Prevax, prevaccination. *P < 0.05, by paired 2-tailed *t* test. Data are shown as the mean ± SEM. Illustration credit: Jennifer Fairman.

To test the translational potential of this therapeutic vaccination strategy, we sought to investigate the immunogenicity of the IN *Mip3a/relMtb* fusion vaccine in rhesus macaques, whose immune system closely resembles that of humans (31–36). We administered the vaccine in 3 doses, 3 weeks apart, at a dose of 1 mg IN per macaque, and measured the RelMtb-stimulated T cell responses before vaccination and 9 weeks and 24 weeks after vaccination (3 and 18 weeks after the last vaccination, Figure 8A). IN immunization with the *Mip3a/relMtb* fusion vaccine led to a relative or statistically significant increase in the percentages of RelMtb-stimulated IFN-γ–, TNF-α–, or IL-17A–producing- CD4^+^ and CD8^+^ T cells in PBMCs and CD4^+^ T cells in BAL at 9 weeks compared with prevaccination measurements (Figure 8B). Each of these immune parameters was found to be inversely correlated with the lung mycobacterial burden in our mouse TB challenge studies (Supplemental Figure 6). While the specific correlates of protection in nonhuman primates with this vaccine remain to be defined, these findings demonstrate that the vaccine generated immune responses in this gold-standard preclinical model that are consistent with the protective responses observed in mouse models, supporting further translational evaluation (31–36). Notably, these responses did not decline substantially 24 weeks after the primary vaccination (Supplemental Figure 11).

## Discussion

In this study, we evaluated a persister-targeting therapeutic vaccination strategy involving IN delivery of a *Mip3a/relMtb* fusion vaccine across multiple preclinical models, including immunocompetent C57BL/6 mice, C3HeB/FeJ mice (which develop human-like lung pathology), and nonhuman primates. The vaccine, when combined with either drug-sensitive or drug-resistant TB regimens, accelerated bacterial clearance without relapse, reduced lung inflammation, and elicited durable TB-protective immune responses. These findings support the potential of this TB therapeutic strategy in clinical settings.

In the setting of TB disease, DCs are fewer and dysfunctional (37–39), leading to altered cellular responses. In our vaccination strategy, we fused the chemokine *Mip3a/CCL20* with the *M. tuberculosis* antigen of interest, RelMtb (13), to target it more efficiently to DCs via the CCR6 receptor in an effort to enhance the crucial crosstalk between innate and adaptive immune responses against *M. tuberculosis* and to optimize cross-presentation to CD4^+^ and CD8^+^ T cells (40), which are essential components of immunity for optimal *M. tuberculosis* control (26, 41). Acknowledging the foremost hurdle to develop not only an effective but also a durable therapeutic vaccine against pulmonary TB, we chose to administer this fusion vaccine via the IN route on the basis of extensive literature showing that respiratory mucosal immunization induces long-lasting, local antigen-stimulated, *M. tuberculosis*–protective T cell responses in contrast to parenteral administration routes (39, 42, 43). Indeed, in the current study, IN immunization with our fusion vaccine enhanced TB control and shortened the duration of curative TB treatment, increased DC recruitment and activation, and elicited robust antigen-stimulated, TB-protective T cell responses systemically and locally compared with RHZE alone. Interestingly, these robust immune responses were sustained, if not augmented, over time.

BPaL is a potent TB drug regimen that was recently recommended by the WHO for DR-TB treatment in the context of the ZeNiX trial, with cure rates of greater than 90% when BPaL was administered for only 6 months with a lower, nontoxic dose of L (29). However, isolates resistant to B and Pa have been increasingly reported, highlighting the potential for spreading drug resistance during real-world implementation (44, 45). Thus, DR-TB is another compelling motivation to develop effective host-directed therapies against TB. Our study shows that the IN *Mip3a/relMtb* fusion vaccine combined with BPaL was more effective in reducing the mycobacterial burden than BPaL alone, suggesting that its adjunctive host-directed therapeutic activity was independent of drug resistance and offering hope that it may also be effective in shortening curative treatment periods for DR-TB.

DNA vaccines have multiple advantages, including cost-efficient production, ease of manufacturing, safety in handling, and a long shelf-life, all compelling features, especially for developing countries where TB is endemic (46). The challenge of their limited immunogenicity in nonhuman primates and humans has historically undermined their use, but recent developments in the context of the COVID-19 pandemic have shed more light on the scientific value of DNA vaccines, revealing several of these to be safe, immunogenic, and highly effective (47–49). Indeed, in this current work, apart from the therapeutic efficacy in mouse models, we found that our DNA IN fusion vaccine was immunogenic in nonhuman primates. One possible explanation of its effective delivery and immunogenicity is the fusion with the Mip3a chemokine, which has been shown to be crucial in driving DC recruitment to the nasal mucosa (50).

The mechanistic basis for this enhanced immunogenicity warrants further discussion. The therapeutic efficacy of our *Mip3a/relMtb* fusion vaccine likely involves coordinated DC recruitment and antigen trafficking. The Mip3a component functions as a CCR6 ligand that recruits immature DCs to sites of antigen expression while simultaneously facilitating antigen uptake. Following IN DNA delivery, vaccine-encoded antigen is expressed across multiple respiratory compartments including nasal epithelium, nasal-associated lymphoid tissue, and conducting airways, where recruited DCs may sample antigen via transepithelial dendrites before migrating to draining LNs (51, 52). Our observation of organized DC–T cell spatial proximity could imply the possible formation of tertiary lymphoid structures (TLSs), which have been documented in TB lung pathology and could represent sites of sustained local immunity. Whether T cell priming occurs primarily through conventional lymph node pathways, local lung-associated lymphoid tissue, or induced TLSs remains an important question for future investigation. Definitive mapping of antigen expression sites, characterization of DC trafficking pathways, and assessment of potential TLS formation through multiplex immunofluorescence and spatial analysis represent high-priority mechanistic studies that will advance our understanding of mucosal DNA vaccine function in tuberculosis immunotherapy.

A limitation of the C3HeB/FeJ mouse studies is that we assessed vaccine effects on bacterial burden and lung pathology during treatment but did not perform a formal relapse analysis in this model. The C3HeB/FeJ strain develops necrotic, hypoxic granulomas that harbor persistent bacilli requiring extended treatment durations (53) beyond standard RHZE regimens to achieve a bacteriological cure. At the end of standard-duration therapy, most mice in both the vaccine and control groups remained culture-positive, precluding an interpretable relapse assessment. Future studies applying optimized treatment durations for this model will be valuable to definitively assess whether the vaccine enables a relapse-free cure in the context of a more advanced disease state. Also, one other limitation of our study was the absence of *M. tuberculosis* challenge experiments in macaques. Such studies are necessary to confirm the therapeutic efficacy of the vaccine in this gold-standard animal model before advancing to human evaluation.

A further limitation is the lack of a benchmark therapeutic vaccine against which to compare the efficacy of the *Mip3a/relMtb* fusion vaccine. In the current proof-of-concept study, we selected *Mip3α/Esat6* as a platform control vaccine to isolate the antigen variable and directly test our central hypothesis: that targeting the persistence-phase, stringent-response antigen RelMtb is superior to targeting an immunodominant replication-phase antigen. This approach builds on prior work demonstrating declining Esat6-reactive and increasing RelMtb-reactive CD4^+^ T cell responses during H therapy (3). Nucleic acid–based vaccines targeting Esat6 have shown variable efficacy in the preventive setting, with one recent systematic screen of 42 antigens showing that an *Esat6* DNA vaccine does not confer significant prophylactic protection compared with several more protective constructs (54), although *Esat6*-dominant DNA platforms have been shown to be protective in mice when formulated to enhance antigen presentation (54–59). Future studies should directly address the question of whether RelMtb is the optimal *M. tuberculosis* persistence antigen for immunotherapeutic targeting as compared with other proteins highly expressed by nonreplicating bacilli, such as those of the DosR regulon (60) as well as other immunodominant antigens, such as the Ag85 complex (61, 62). In addition, the efficacy of the *Mip3α/relMtb* fusion vaccine should be tested against other therapeutic vaccines, such as RUTI, or those targeting other latency antigens, such as Rv2660c (63–65).

Finally, this study does not fully delineate how standard chemotherapy alone augments RelMtb-stimulated T cell responses. Prior H monotherapy studies demonstrated increases in RelMtb-stimulated CD4^+^ T cells compared with untreated controls (3), whereas in the context of the first-line regimen studied here, only modest, nonsignificant increases were seen with RHZE alone, and robust responses occurred only with *Mip3a/relMtb* vaccination, underscoring the need for studies explicitly mapping RelMtb antigen availability and T cell dynamics across drug regimens, with designs powered to separate chemotherapy and vaccine effects.

In conclusion, this study provides a paradigm shift toward the development of safe therapeutic DNA TB vaccines focused on shortening TB drug treatment and decreasing relapse rates in various settings, including for both drug-susceptible and DR-TB without promoting drug resistance or adverse drug reactions. The vaccine’s success in eliciting durable, TB-protective immunity underscores its translational potential for clinical use. The data also support the successful delivery of this DNA vaccine not only in small animal models but also in nonhuman primates, making it a promising vaccine for successful clinical application. Furthermore, these findings establish a framework for defining immune correlates of TB control, emphasizing the critical role of DC–T cell crosstalk and antigen-stimulated responses in eradicating persistent infection. This approach paves the way for accelerated therapeutic vaccine development to address unmet needs in global TB management.

## Methods

### Sex as a biological variable.

Our study examined male and female animals, and similar findings are reported for both sexes.

### Mouse M. tuberculosis infection studies.

Four- to 6-week-old male and female C57BL/6 and C3HeB/FeJ mice were purchased from The Jackson Laboratory. C57BL/6 experiments included 5–20 mice per group; C3HeB/FeJ experiments included 19–55 mice per group. The mice were housed in individually ventilated cages and maintained on a 12-hour light/12-hour dark cycle with free access to food and water. They were monitored at least weekly, with recording of their weight and assessment of their general appearance. The mice were infected between 6 and 8 weeks of age via the aerosol route using the Glas-Col Inhalation Exposure System. A fresh aliquot of WT *M. tuberculosis* H37Rv, gifted by Sandeep Tyagi (Johns Hopkins University, Baltimore, Maryland, USA), was used for each infection and diluted in 7H9 broth (Difco) with 10% oleic acid–albumin–dextrose–catalase (OADC) and glycerol with or without Tween-80 to achieve the desired inoculum per experiment (3). The CFU implantation goal for the chronic TB infection model was approximately 2 log_10_ *M. tuberculosis* bacilli; for the subacute TB infection model, the goal was approximately 4 log_10_. To reduce intergroup variability, mice assigned to the same experiment were infected together or evenly distributed between infection cycles and randomly assigned to each group per experiment. On the day after infection, at least 5 mice per experiment were sacrificed to determine the number of CFU implanted in the lungs.

### Mouse treatments.

After 14 (C57BL6/BPaL experiment), 28 (C57BL6/RHZE experiments), or 56 (C3HeB/FeJ/RHZE experiment) days of infection, at least 5 animals per experiment were sacrificed to determine the number of CFU present at the start of treatment. On the same day, for the RHZE experiments in C57BL6 and C3HeB/FeJ mice, the mice were randomized to receive human-equivalent doses of H (10 mg/kg), R (10 mg/kg), Z (150 mg/kg), and E (100 mg/kg) once daily dissolved in distilled water or to receive distilled water only (control group) (66). The Z solution was gently heated in a 55°C water bath and vortexed to dissolve before treating the mice. To minimize drug-drug interactions, R was administered at least 1 hour before Z. For the BPaL experiment in C57BL/6 mice, the mice were randomized to receive B (25 mg/kg, formulated in 20% hydroxypropyl-β-cyclodextrin solution acidified with 1.5% 1N HCl) (67), Pa (50 mg/kg, prepared in the CM-2 formulation) (68), and L (50 mg/kg, prepared in 0.5% methylcellulose) (67) once daily or vehicle mix only (control group). The BPaL doses were selected to match the optimal human doses to minimize toxic effects while maintaining efficacy (29). To minimize drug-drug interactions, L was administered 4 hours after BPa. All drugs in all experiments were administered as indicated, once daily, by oral gavage, in a total volume of 0.2–0.3 mL/mouse daily.

### DNA vaccine construct design and gene nomenclature.

Throughout this article, we use the nomenclature *relMtb* (or RelMtb for the protein) to refer specifically to the Mtb *relA* gene (systematic designation Rv2583c (69)), which encodes the bifunctional (p)ppGpp synthetase/hydrolase (70). The plasmid pSectag2B, encoding the full-length *relMtb* gene (Rv2583c), served as the basis for DNA vaccine construction (3, 13). Similarly, *Esat6* is used throughout the article to denote the early secreted antigenic target of 6 kDa protein, encoded by the *esxA* gene (systematic designation Rv3875).

The *relMtb* gene was codon optimized for mammalian expression (GenScript) and fused in-frame to the mouse *Mip3α* gene.(13) This fusion construct was cloned into pSectag2B to generate the *Mip3α/relMtb* vaccine (13). Correct insertion was confirmed by DNA sequencing (Johns Hopkins University DNA Sequencing Facility, Baltimore, Maryland, USA) (13). Target gene expression was validated by transient transfection of HEK-293T cells (ATCC) using Lipofectamine 2000 (Invitrogen, Thermo Fisher Scientific), with protein detection in both cell lysates and culture supernatants confirmed by Western blot analysis using anti-His antibodies (MilliporeSigma) (13). Vaccination plasmids were propagated in *E. coli* DH5α (Invitrogen, Thermo Fisher Scientific), selected with ampicillin (100 μg/mL), and purified using Qiagen EndoFree Plasmid Kits according to the manufacturer’s instructions. Plasmids were resuspended in endotoxin-free PBS (1×) (13). An analogous *Mip3α/Esat6* vaccine construct was produced using identical methodology.

### Mouse vaccinations.

At the time of drug treatment initiation, mice were randomized to receive the *relMtb* vaccine or the fusion *Mip3a/relMtb* vaccine by the IM (10 μg in 50 μL in each quadriceps, followed by electroporation) or IN (100 μg in 50 μL in each nostril) route as described previously (13). For the control or drug-only treatment groups, the mice received mock vaccination with IN PBS (50 μL per nostril per mouse). The mice were vaccinated 3 times at 1-week intervals. IM or IN delivery of each DNA plasmid followed adequate anesthesia of mice by vaporized isoflurane (13).

### Fluid and tissue collection and bacterial enumeration.

Mice were sacrificed 6, 8, 10, 12, or 24 weeks after drug treatment and/or immunization series. For the efficacy time points and experiments, half spleens, right lower lungs, and LNs were harvested and processed into single-cell suspensions after mechanical disruption and filtration through a 70 μm cell strainer. Lung tissue was additionally digested using collagenase type I (Thermo Fisher Scientific), and DNase (MilliporeSigma) for 20 minutes at 37°C (13). Whole blood was collected through terminal cardiac puncture and processed into PBMCs (13). BAL was performed, fluid was collected, and cellular components were isolated as a single-cell suspension (71). Single-cell suspensions were resuspended in warm R10 (RPMI 1640 with 2 mM glutamine, 100 U/mL penicillin, 100 μg/mL streptomycin, and 10% heat-inactivated FBS; Atlantic Biologicals) for flow cytometric analysis as detailed below. The right upper lung from each mouse was homogenized using glass homogenizers. CFU were normalized to tissue weight to establish a standardized metric independent of the tissue sampled. Serial 10-fold dilutions of lung homogenates in PBS were plated on 7H11 selective agar (BD) for CFU enumeration. Plates were incubated at 37°C, and CFU were counted 4 and 6 weeks later by at least 2 investigators (13). Left whole lung was fixed by immersion in 10% neutral buffered formalin for at least 48 hours, paraffin embedded, and sectioned, followed by quadruple immunolabeling for DAPI^+^CD45^+^CD3^+^CD11c^+^F4/80^+^ or H&E and staining of acid-fast bacilli (Ziehl-Neelsen), as detailed below. For the relapse time point (24-week time point), whole spleens and whole lungs were plated for CFU enumeration.

### Macaque vaccinations, procedures, and sample collection.

One- to 3-year-old male and female rhesus macaques purchased from the Johns Hopkins University Breeding Farm were used in the immunogenicity study. All macaques were housed in single-species harem breeding groups or same-sex juvenile/young adult groups. Animal enclosures consisted of runs with concrete flooring or raised corncrib cages. All macaques had indoor and outdoor access, were fed a standard commercial diet (rhesus macaques: 5049 Fiber-Plus Monkey Diet, LabDiet) and rotating food enrichment items including fresh fruits, vegetables, and dried fruit treats. Animals were provided water ad libitum. Annual colony health screening included intradermal tuberculin testing and serology for macacine herpesvirus 1 (B virus), simian immunodeficiency virus, simian T cell leukemia virus, and simian retrovirus. All animals were consistently negative on TB testing and viral serology. Macaques were sedated with 10 mg/kg ketamine IM to receive IN immunization with the fusion *Mip3a/relMtb* vaccine. Animals were monitored closely throughout sedation until complete recovery. The vaccine was delivered as 500 μg plasmid DNA in 250 μL dripped into each nostril via pipette. Each macaque received 3 vaccinations administered at 3-week intervals (weeks 0, 3, and 6). Before prime immunization, 9 weeks and 24 weeks after the final vaccination, veterinary staff collected BAL fluid and whole blood. BAL fluid was collected after ketamine and dexmedetomidine sedation and intubation. A single-use pediatric suction catheter (Airlife Tri-Flo Suction Catheter with Control Port, Carefusion) was inserted through the endotracheal tube connector and blindly passed through the trachea and into a mainstem bronchi. The catheter was passed until resistance was felt, indicating the catheter had wedged into a distal bronchus. A 20 mL syringe containing the lavage fluid was attached to the end of the catheter and infused over 1–2 seconds. Immediately after the infusion, the aliquot was manually aspirated into the syringe using gentle pulsating suction. This process was repeated 2–3 times per animal. After the procedure, anesthesia was reversed with atipamazole hydrochloride (Antisedan, Zoetis), and all animals were extubated and allowed to recover. Blood draws of 4 mL were obtained at the points of BAL (under sedation). BAL samples were processed into BAL single-cell suspensions by centrifugation and blood samples into PBMCs by Ficoll-Paque PLUS gradient separation (GE Healthcare Biosciences) for flow cytometric analysis.

### Recombinant RelMtb protein preparation.

The previously generated *rel*Mtb expression plasmid pET15b[*rel*Mtb] (3, 13, 17, 70) was used for expression and purification of recombinant RelMtb protein. *E. coli* BL21(DE3)RP cells (Stratagene) were transformed and selected with ampicillin (100 μg/mL). Protein expression was induced with 0.5 mM IPTG at 37°C for 4 hours. Recombinant His-tagged RelMtb protein (87 kDa) was purified from bacterial lysate using Ni-NTA Agarose columns (QIAexpress Ni-NTA Fast Start System-Qiagen, catalog 30600) followed by 1% Triton X-114 washing (MilliporeSigma, T6009) in an effort to solubilize and remove LPS (72). Protein purity was assessed by SDS-PAGE followed by Coomassie brilliant blue staining and showed a predominant band at the expected molecular weight (~80 kDa) with minimal contaminating bands. Identity was confirmed by immunoblotting. Protein concentrations were determined by bicinchoninic acid (BCA) assay (Thermo Fisher Scientific). Purified protein was aliquoted and stored at –80°C. Protein aliquots were thawed only once to maintain structural integrity and minimize aggregation. For ex vivo T cell stimulation assays, 1 × 10^6^ cells/well were cultured with 10 μg/mL recombinant RelMtb for 12–24 hours at 37°C and 5% CO_2_. Controls included unstimulated cells. Recombinant RelMtb retains (p)ppGpp synthesis/hydrolysis activities (3, 70).

### Multiparameter flow cytometry.

Single-cell suspensions from spleens, lungs, draining LNs, BAL, and PBMCs derived from mice or macaques were prepared as described above. For LNs, BAL, and PBMCs, samples were pooled into 2–7 samples to achieve at least 10^5^ alive cells per sample. When applicable, each tissue was stimulated with purified recombinant RelMtb protein at 37°C (3, 19) for various time intervals, from 12 hours (IFN-γ, IL-17A) to 24 hours (TNF-α), depending on the cytokine of interest (13). For intracellular cytokine staining (ICS), GolgiPlug cocktail (BD Pharmingen) was added for an additional 4 hours after stimulation (total, 16 and 28 hours, respectively). Cells were collected using FACS buffer (PBS plus 0.5% BSA (MilliporeSigma), stained with Zombie NIR Fixable Viability Kit (BioLegend, catalog 423106) for 30 minutes, and then washed with PBS buffer. Surface proteins were stained for 20 minutes, the cells were fixed and permeabilized with buffers from the BioLegend intracellular fixation/permeabilization set following the manufacturer’s protocols (catalog 421002), intracellular proteins were stained for 20 minutes, and samples were washed and resuspended with FACS buffer. The following anti-mouse mAbs were used: PE-conjugated anti-CD45 (BioLegend, catalog 103106); BV650-conjugated anti-CD45 (BioLegend, catalog 103151); BV421-conjugated anti-CD45 (BioLegend catalog 103134), PerCPCy5.5 conjugated anti-CD3 (BioLegend, catalog 100218); PE/Dazzle 594–conjugated anti-CD4 (BioLegend, catalog 100566); Alexa Fluor 700–conjugated anti-CD8 (BioLegend, catalog 100730); PECy7-conjugated anti–TNF-α (BioLegend, catalog 506324); APC-conjugated anti–IFN-γ (BioLegend, catalog 505810); PE-conjugated anti–IL-17A (BioLegend, catalog 506904); BUV395-conjugated anti–IL-17A (BD Biosciences, catalog 565246); BUV563-conjugated anti-CD19 (BD Biosciences, catalog 749028); BUV496-conjugated anti–NK-1.1 (BD Biosciences, catalog 741062); BV605-conjugated anti-Ly6G (BioLegend, catalog 127639); BV421-conjugated anti-CD11c (BioLegend, catalog 117330); PE-conjugated anti-CD11c (BioLegend, catalog 117308), BUV395-conjugated anti-CD24 (BD Biosciences, catalog 744471); BUV805-conjugated anti-CD11b (BD Biosciences, catalog 741934); BV785-conjugated anti-XCR1 (BioLegend, catalog 148225); BV650-conjugated anti-CD80 (BioLegend, catalog104732); BV510-conjugated anti–MHC II (BioLegend, catalog 107636); and FITC-conjugated anti-CD103 (BioLegend, catalog 121420). The following anti–nonhuman primate or human antibodies were used: FITC-conjugated anti-CD3 (Thermo Fisher Scientific, catalog APS0308); Alexa Fluor 700–conjugated anti-CD4 (Thermo Fisher Scientific, catalog 56-0048-41); APC anti-CD8 (Thermo Fisher Scientific, catalog MA5-44089); PerCPCy5.5–conjugated anti–TNF-α (Thermo Fisher Scientific, catalog 45-7349-42); PE-Cy7–conjugated anti–IFN-γ (Thermo Fisher Scientific, catalog 25-7319-82); and PE-conjugated anti–IL-17A (Thermo Fisher Scientific, catalog 12-7178-42). The Attune NxT (Thermo Fisher Scientific), BD, and BD Biosciences LSR II/Fortessa flow cytometers were used. Flow data were analyzed by FlowJo Software 10.8.1. Gating strategies can be found in Supplemental Figures 12 and 13. DC gating was performed according to methods established in previous literature (73).

### Luminex multiplex cytokine analysis.

BAL fluid was collected from *M. tuberculosis*–infected mice 6 weeks after the prime vaccination. BAL supernatants were separated from cellular components by centrifugation, filtered through 0.22 μm PVDF membrane filters, and stored at –80°C until analysis. The Bioplex 200 platform (Bio-Rad) was used to determine the concentration of secreted cytokine levels (TNF-α, IL-17A, IFN-γ). Luminex bead–based immunoassays (MilliporeSigma) were performed by the Sidney Kimmel Cancer Center Immune Monitoring Core at Johns Hopkins following validated test methods. Concentrations were determined using 5-parameter log curve fits (using Bioplex Manager 6.0) with vendor-provided standards and quality controls. Concentrations that were outside of the standard curve values were categorized as out of range (OOR). For each cytokine, values below the OOR value were replaced with the lower limit of the standard curve of the assay.

### Lung histopathology.

Left lung lobes were harvested from C3HeB/FeJ mice 20 weeks after infection (12 weeks after treatment initiation) to capture well-developed granulomas. Formalin-fixed tissues were sectioned and stained with H&E. Stained sections were imaged at ×40 and ×200 magnification. Lesion quantification included (a) the ratio of lymphohistiocytic aggregate area (with/without necrosis) to total lung area per slide and (b) the average inflamed area of inflammatory foci per animal. Quantification was performed by a blinded certified pathologist using QPath, with results visualized in GraphPad Prism (GraphPad Software). For AFB detection, the Ziehl-Neelsen method was applied. Stained sections were imaged at ×40 and ×600 magnification. Because of the scarcity of AFB^+^ bacilli in both the RHZE and vaccine-plus-RHZE groups, AFB quantification was not performed.

### Immunofluorescence.

Quadruple immunolabeling for CD45^+^CD3^+^CD11c^+^F4/80^+^ was performed on formalin-fixed, paraffin-embedded sections derived from *M. tuberculosis*–infected C57BL6 mouse lungs (left lobe) on a Ventana Discovery Ultra autostainer (Roche Diagnostics). Following dewaxing and rehydration on board, epitope retrieval was performed using Ventana Ultra CC1 buffer (Roche Diagnostics, catalog 6414575001) at 96^o^C for 64 minutes. Anti-CD45 primary antibody (1:200 dilution; Cell Signaling Technology, catalog 702575S) was applied at 36°C for 40 minutes. Primary antibody was detected using an anti-rabbit HQ detection system (Roche Diagnostics, catalogs 7017936001 and 7017812001) followed by OPAL 520 (Akoya Biosciences, NEL871001KT) diluted 1:150 in 1× Plus Amplification Diluent (Akoya Biosciences, catalog FP1498). Following CD45 detection, primary and secondary antibodies from the first round of staining were stripped on board using Ventana Ultra CC1 buffer at 95°C for 12 minutes followed by neutralization using Discovery Inhibitor (Roche Diagnostics, catalog 7017944001). Anti-CD3 primary antibody (1:200 dilution; Abcam, catalog ab16669) was applied at 36°C for 40 minutes. Anti-CD3 primary antibodies were detected using an anti-rabbit HQ detection system (Roche Diagnostics, catalogs 7017936001 and 7017812001) followed by OPAL 570 (Akoya Biosciences, NEL871001KT) diluted 1:150 in 1× Plus Amplification Diluent (Akoya Biosciences, catalog FP1498). Primary and secondary antibodies from the second staining round were stripped on board using Ventana Ultra CC1 buffer at 95^°^C for 12 minutes, followed by neutralization using Discovery Inhibitor (Roche Diagnostics, catalog 7017944001). Anti-CD11c primary antibody (1:300 dilution; Thermo Fisher Scientific, catalog PAS-79537) was applied at 36°C for 40 minutes. CD11c primary antibodies were detected using an anti-rabbit HQ detection system (Roche Diagnostics, catalogs 7017936001 and 7017812001) followed by OPAL 690 (Akoya Biosciences, NEL871001KT) diluted 1:150 in 1× Plus Amplification Diluent (Akoya Biosciences, catalog FP1498). Following CD11c detection, primary and secondary antibodies from the third staining round were stripped on board using Ventana Ultra CC1 buffer at 95°C for 12 minutes and neutralization with Discovery Inhibitor (Roche Diagnostics, catalog 7017944001). Anti-F4/80 primary antibody (1:1,000 dilution; Cell Signaling Technology, catalog 70076) was applied at 36°C for 40 minutes. F4/80 primary antibodies were detected using the anti-rabbit HQ detection system (Roche Diagnostics, catalogs 7017936001 and 7017812001) followed by OPAL Polaris 780 (Akoya Biosciences, NEL871001KT) diluted 1:150 in 1× Plus Amplification Diluent (Akoya Biosciences, catalog FP1498). Tissue was then counterstained with spectral DAPI (Akoya Biosciences, FP1490) and mounted with Prolong Gold (Thermo Fisher Scientific, P36930).

All slides were scanned using the FL Zeiss whole-slide scanner at ×40 resolution. Images were initially viewed using Zeiss Zen Lite software; the HALO platform version 3.4.2986 (Indica Labs) was used for quantitative analysis using the HighPlex FL algorithm. Cell-cell interactions were assessed using proximity thresholds of 10 μm (approximating cell diameter) for standard spatial analysis and 5 μm to identify even closer proximity interactions. Cells with center-to-center distances at or below the respective threshold were considered to be interacting.

### Statistics.

All reported *P* values were determined by 2-sided comparisons. Pairwise comparisons of group mean values for CFU (microbiology data), flow cytometric data, and Luminex, histopathology, and immunofluorescence data were done using 1-way ANOVA followed by Tukey’s multiple-comparison test, 2-tailed *t* test, or Mann-Whitney *U* test where indicated. Several simple linear regressions were performed to assess the correlation of each individual immune response per tissue expressed in percentages (independent variable) with lung mycobacterial outcome (log_10_ transformed–dependent variable). The *R^2^* value was set above 0.6 to ensure linear correlation, and the *P* value was set at less than 0.05. GraphPad Prism 10.2.0 (GraphPad Software) was utilized for statistical analyses and graph generation. All error bars represent the estimation of the SEM, and all midlines represent the group mean unless otherwise specified. In CFU data visualization, we did not use logarithmic transformation when mice had undetectable lung mycobacterial burden due to the mathematical undefined nature of log_(0)_. A significance level of α ≤ 0.05 was set for all experiments.

### Study approval.

All animal studies were performed per the protocols approved by the Johns Hopkins Animal Care and Use Committee of the Johns Hopkins School of Medicine. Macaques were housed and cared for following local, state, federal, and institutional policies in facilities accredited by the American Association for Accreditation of Laboratory Animal Care (AAALAC) under standards established in the Animal Welfare Act and the Guide for the Care and Use of Laboratory Animals. Macaques were monitored for physical health, food consumption, body weight, and temperature. All experiments with *M. tuberculosis* in mice were conducted in Institutional Biosafety Committee–approved BSL3 and ABSL3 facilities at The Johns Hopkins University School of Medicine using recommended positive-pressure air respirators and protective equipment.

### Data and materials availability.

All data are available in the Supporting Data Values file.

## Author contributions

SK, RBM, and PCK conceptualized the study. SK, RBM, PCK, EN, TK, JDP, TW, RT, JTG, and ARM designed the study methodology. SK, TK, JDP, TW, RT, AY, JRC, JTG, HB, DQ, FS, RBM, KF, FC, HTH, EMRS, REB, ADT, YL, JM, HT, JJZ, and JZ performed experiments. SK, TW, TK, JDP, and FS conducted visualization. SK, EN, RBM, and PCK acquired funding. SK, RBM, and PCK handled project administration. SK, RBM, and PCK supervised the work. SK wrote the original draft of the manuscript. SK, RBM, PCK, EN, TK, JDP, TW, AY, JRC, JTG, HB, DQ, RT, FS, RM, KF, FC, HTH, ERS, REB, ADT, YL, JM, HT, JJZ, and JZ reviewed and edited the manuscript.

## Funding support

This work is the result of NIH funding, in whole or in part, and is subject to the NIH Public Access Policy. Through acceptance of this federal funding, the NIH has been given a right to make the work publicly available in PubMed Central.

- NIH grant R01AI148710 (to PCK and RBM)
- NIH grant K24AI143447 (to PCK)
- NIH grant P30AI18436 (to PCK and EN)
- NIH grant K08AI174959 (to SK)
- NIH grant P30CA006973 (to William Nelson, Cancer Center Core, Johns Hopkins University)
- Gilead HIV Research Scholar Award (to SK)
- Tuberculosis Research Advancement Center Developmental Award, Johns Hopkins University, P30AI168436 (to SK)
- Center for HIV/AIDS Developmental Award, Johns Hopkins University Center for AIDS Research, P30AI094189 (to SK)
- Willowcroft Foundation Award (to SK)
- Clinician Scientist Award, Johns Hopkins University (to SK)
- Potts Memorial Foundation (to SK)

## Supplementary Material

Supplemental data

Supporting data values

## Version 1 02/03/2026

In-Press Preview

## Version 2 04/01/2026

Electronic publication
