# Supplementary material for: Immunotherapy targeting drug-tolerant Mycobacterium tuberculosis persisters accelerates tuberculosis cure in preclinical models
Source: J Clin Invest. 2026 Feb 3;136(7):e196648. doi: 10.1172/JCI196648 (PMC13038216; doi:10.1172/JCI196648)
Supplement: Supplemental data [file jci-136-196648-s177.pdf]

Supplementary Figures 1-10

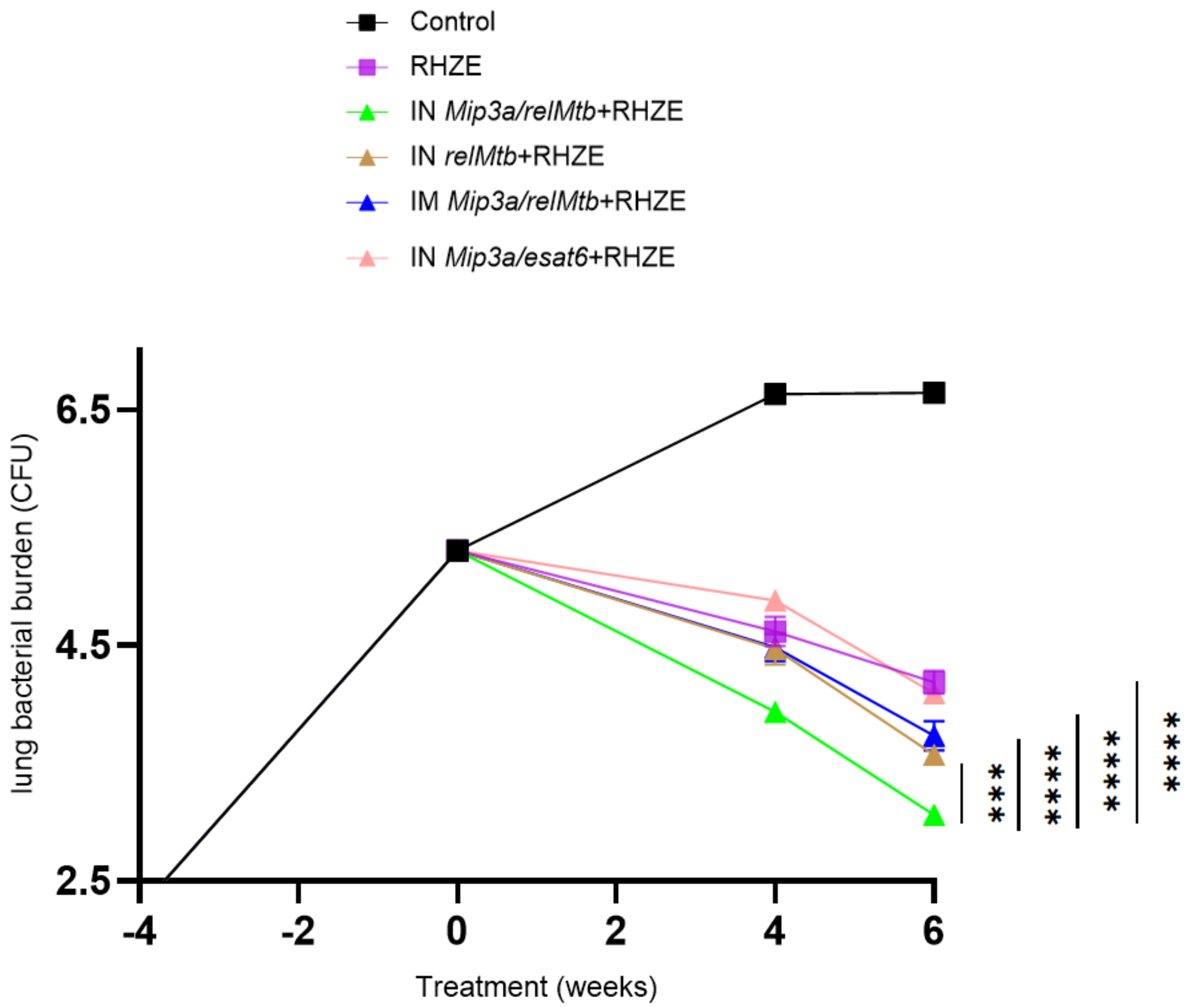

Supplementary Figure 1. Therapeutic IN administration of the *Mip3a/relMtb* fusion vaccine enhances the efficacy of the first-line regimen for drug-susceptible TB in C57BL6 mice. Timeline of mean lung mycobacterial burden at 4 and 6 weeks after the primary vaccination per vaccination group (n=5 mice/group). Mtb, *Mycobacterium tuberculosis*; IM: intramuscular; IN: intranasal; Esat6, Early Secreted Antigenic Target; CFU, colony-forming units; RHZE, Rifampin-Isoniazid-Pyrazinamide-Ethambutol.

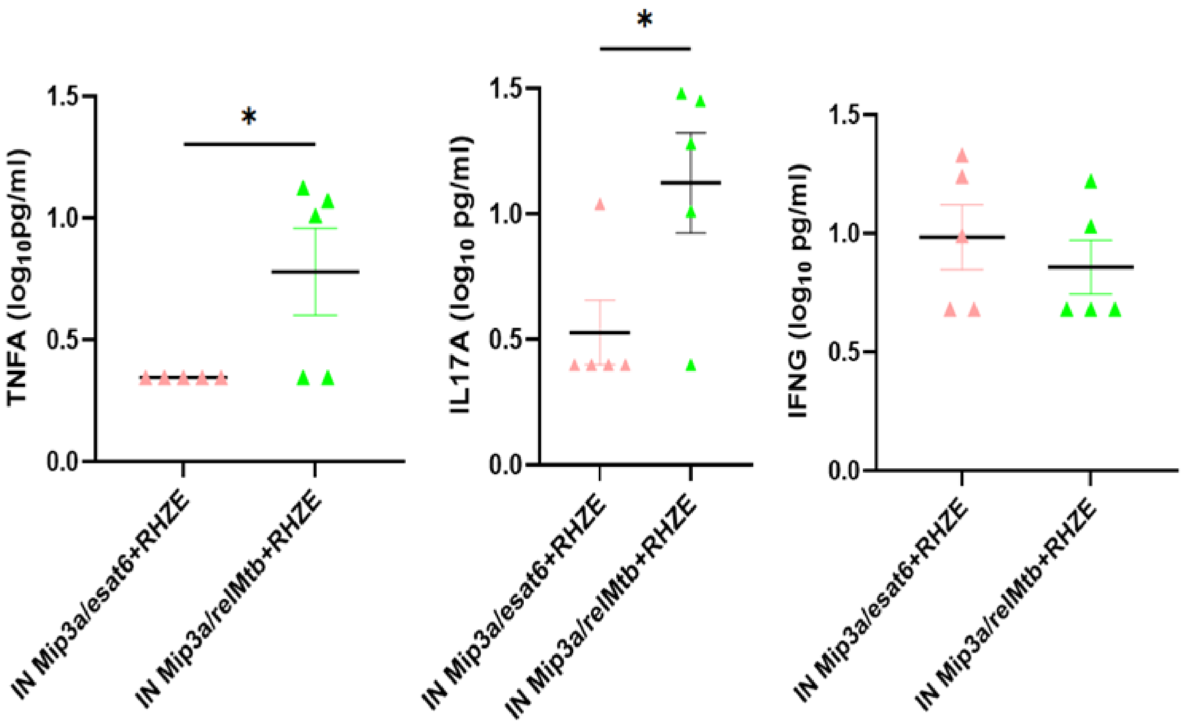

Supplementary Figure 2. Comparative analysis of secreted cytokines in bronchoalveolar lavage (BAL) fluid following intranasal vaccination with *Mip3α/esat6* versus *Mip3α/relMtb*. C57BL/6 mice received intranasal vaccination (3 doses) with either *Mip3a/esat6* or *Mip3a/relMtb* DNA vaccines. Six weeks post- prime vaccination, BAL was performed, and cell-free supernatants were analyzed by Luminex multiplex cytokine assay (BioPlex 200 platform) for TNFA, IL17A, and IFNG. Mice vaccinated with intranasal Mip3α/relMtb exhibited significantly elevated TNFA ($P = 0.0407$) and IL17A ($P = 0.0360$) secretion compared to intranasal *Mip3α/esat6* -vaccinated mice, while IFNG levels were comparable between groups ($P = 0.4965$). Each data point represents an individual mouse (n=5 per group). Cytokine concentrations are expressed as $\log_{10}$ pg/mL. Statistical significance was determined by unpaired t-test. *$P < 0.05$.

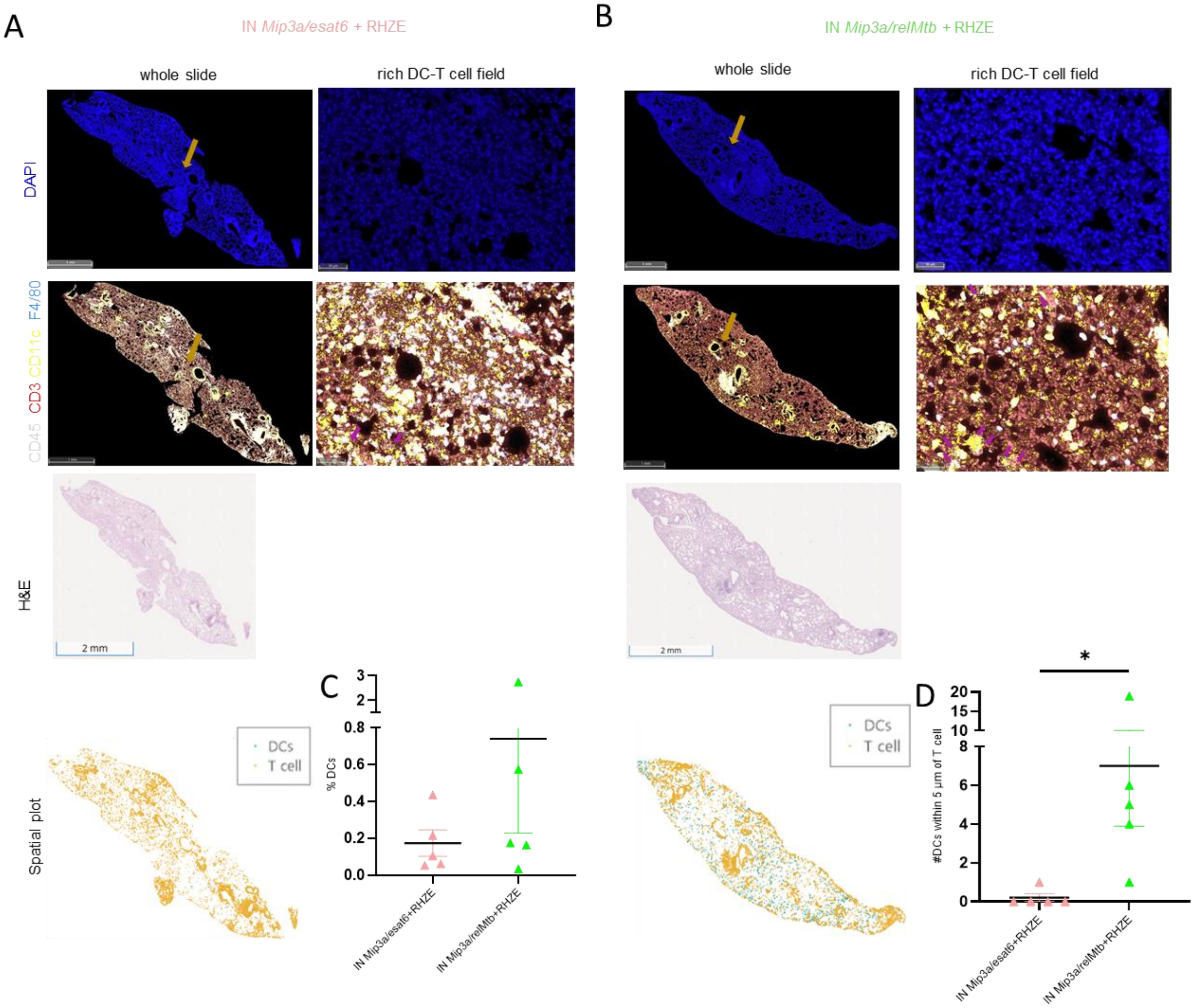

Supplementary Figure 3. Spatial analysis of dendritic cell (DC) recruitment and DC-T cell interactions in lungs of mice following intranasal vaccination with Mip3a/esat6+RHZE versus Mip3a/relMtb+RHZE. A, B. Representative lung sections (whole slides, rich dendritic cell-T cell fields of view and DC-T cell spatial plots) from *Mip3a/esat6*+RHZE (A) vs. IN *Mip3a/relMtb*+RHZE after 6 weeks of treatment (B) stained with antibodies for DAPI+ only (dark blue, cell nuclei, top) or CD45+ (white, hematopoietic cells, bottom), CD45+CD3+ (red, T cells, bottom) and CD45+CD3-CD11c+F4/80- (yellow, dendritic cells, bottom). Light brown arrows in low magnification images indicate the specific areas shown in the corresponding high-magnification images. Magenta arrows indicate DC-T cell colocalization. C, D. Quantification of total dendritic cells per DAPI+ cells (%) (C) and (D) colocalization of dendritic-T cells defined as number of dendritic cells within 5 μm of T cells [*Mip3a/esat6*+RHZE (n=5) vs. *Mip3a/relMtb*+RHZE (n=5), Mann-Whitney test was used, assessment was performed on sections encompassing the entire left lungs]; DCs, dendritic cells; RHZE, Rifampin-Isoniazid-Pyrazinamide-Ethambutol; *P<0.05.

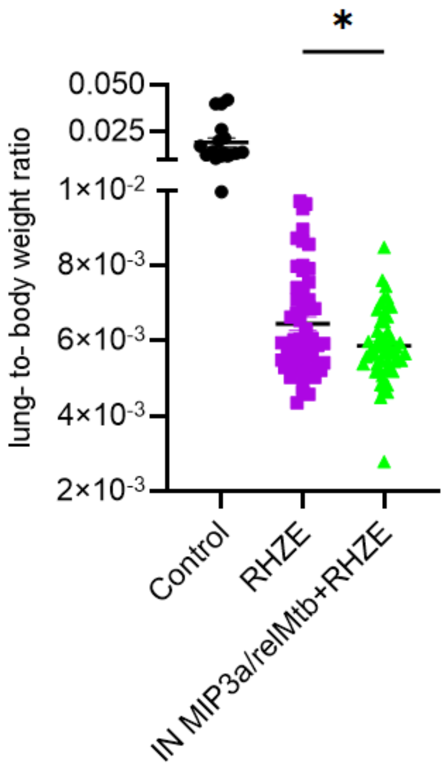

Supplementary Figure 4. The therapeutic *Mip3a/relMtb* fusion vaccine, when combined with the first-line regimen, significantly reduced lung inflammation in C3HeB/FeJ mice compared to drug treatment alone, as evidenced by the improved lung-to-body weight ratios. Scatterplot of the lung to body weight ratio at 12 weeks after RHZE treatment initiation/prime vaccination. Unpaired t-test was used; $n_{Control}$= 19, n_{RHZE}=55, $n_{IN\ Mi3a/relMtb\ \&\ RHZE}$=55; RHZE: Rifampin, Isoniazid, Pyrazinamide, Ethambutol, CFU: colony-forming units, Mtb, *Mycobacterium tuberculosis*; IN: intranasal; CFU, colony-forming units; RHZE, Rifampin-Isoniazid-Pyrazinamide-Ethambutol. *P<0.05.

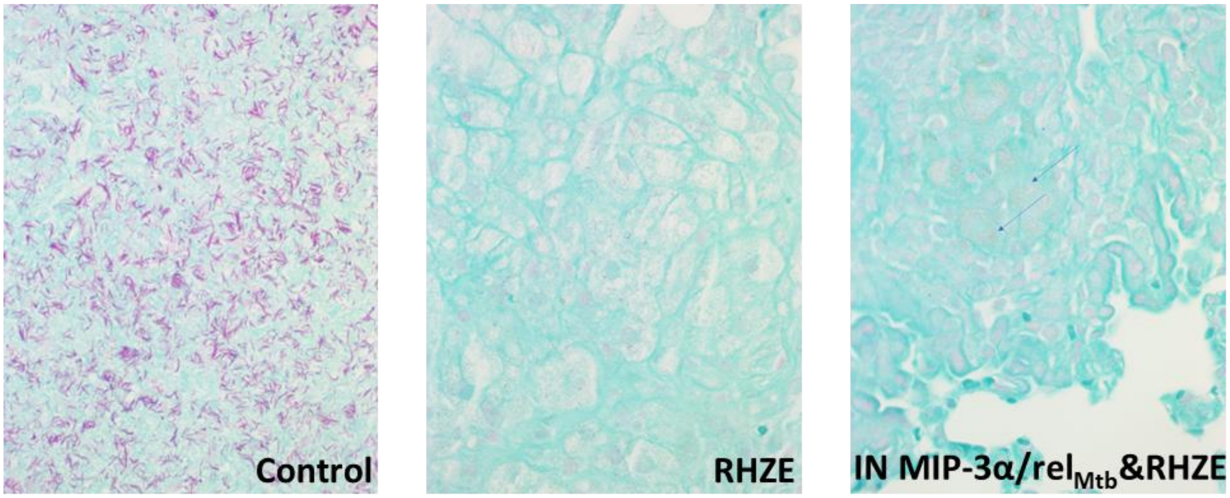

Supplementary Figure 5. AFB stain in C3HeB/FeJ murine lungs. Lungs (left) collected at 12 weeks post treatment initiation and 20 weeks post infection. The control C3HeB/FeJ mice show widespread granulomatous lung inflammation including large central necrosis which corresponded with a very high load of AFB+ bacilli in these necrotic areas whereas the lungs of C3HeB/FeJ mice receiving either RHZE or IN *Mip3a/relMtb* fusion vaccine + RHZE both show foci of reduced inflammation, mostly consistent with scattered lymphocytic infiltrates surrounding foamy histiocytes, without evidence of necrosis with only rare AFB+ bacilli in the lesions, which could not be reliably quantified through pathology. Representative images for each group at 600X; IN: intranasal; Rifampin, Isoniazid, Pyrazinamide, Ethambutol.

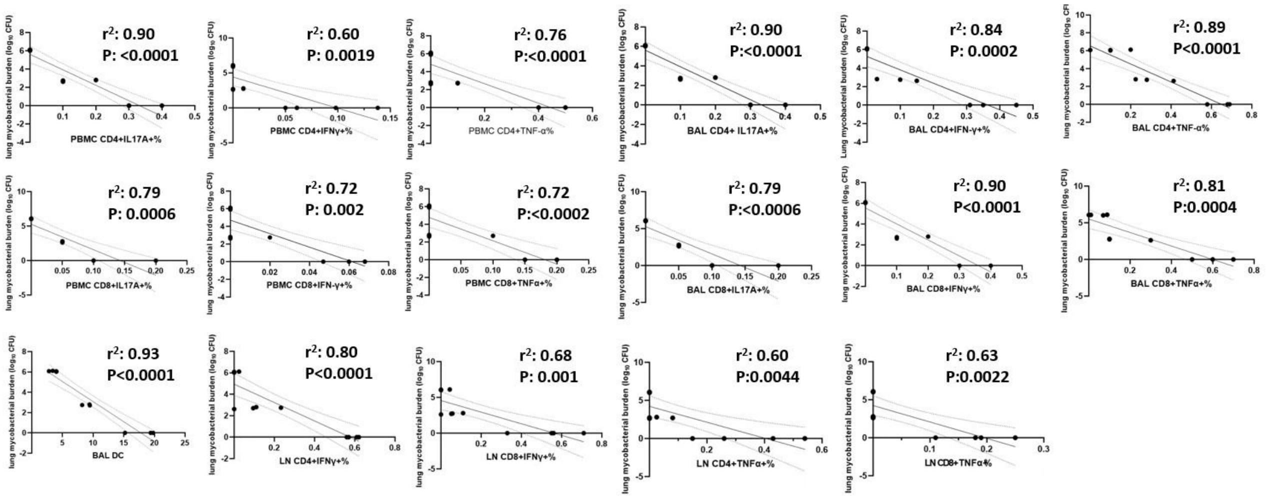

Supplementary Figure 6. Relationships between RelMtb-specific T-cell responses and dendritic cells in PBMC, BAL, and mediastinal LNs and total CFUs at 12 weeks post-prime vaccination and/or initiation of drug treatment in immunocompetent mice. Linear regressions were used to test whether antigen-specific CD4+ or CD8+ T-cell responses (IFNG, TNFA, or IL17A) (%) are associated with lung mycobacterial burden (total CFUs) in spleen, PBMCs, lungs, mediastinal LNs, and BAL. Results indicate that CD4+ and CD8+ T-cell producing IL17A, IFNG, TNFA (%) in PBMCs and BAL along with total DCs (%) in BAL and CD4+ and CD8+ T-cell producing IL17A and TNFA (%) in mediastinal lymph nodes are significantly negatively associated with lung mycobacterial burden. R^2 is the coefficient of determination. Each dot represents the pooled sample (single-cell suspension) from 2-7 animals. Black lines represent linear fit (with 95% confidence interval in dotted black lines). PBMC, peripheral blood mononuclear cells; BAL, bronchoalveolar lavage; LN, lymph nodes; CFU, colony-forming units.

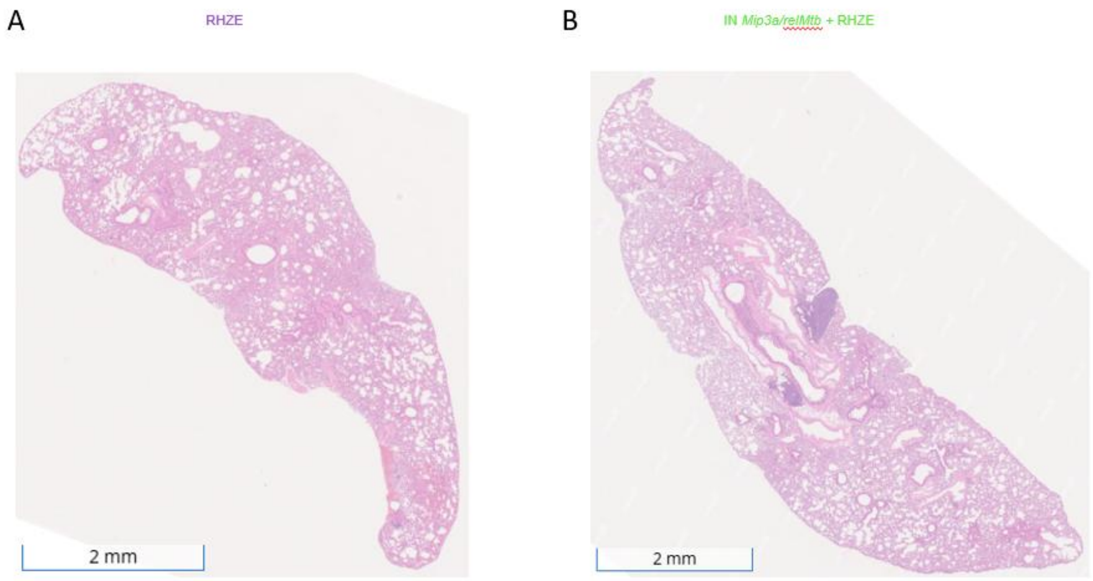

Supplementary Figure 7. Representative H&E lung sections (whole slide). RHZE alone (A) vs. IN *Mip3a/relMtb* + RHZE after 12 weeks of treatment (B). RHZE, Rifampin-Isoniazid-Pyrazinamide-Ethambutol.

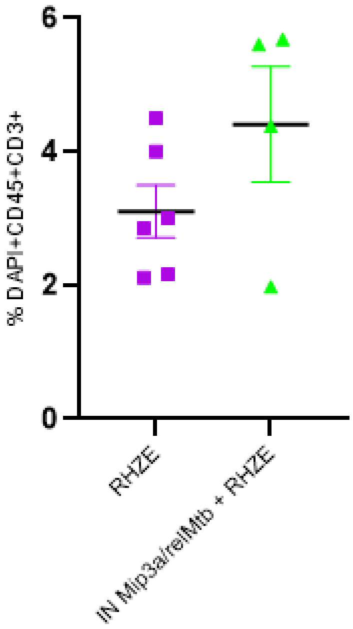

Supplementary Figure 8. T cells in mouse lungs do not statistically differ between RHZE vs. IN *Mip3a/relMtb* + RHZE after 12 weeks of treatment in immunocompetent mice as measured through immunofluorescence. T cells are defined as %DAPI+CD45+CD3+ and quantified as % T cells/ DAPI+ cells. [RHZE (n=6) vs. IN *Mip3a/relMtb* & RHZE (n=4), P=0.352, Mann-Whitney test was used, the assessment was performed on sections encompassing the entire left lungs]; RHZE, Rifampin-Isoniazid-Pyrazinamide-Ethambutol.

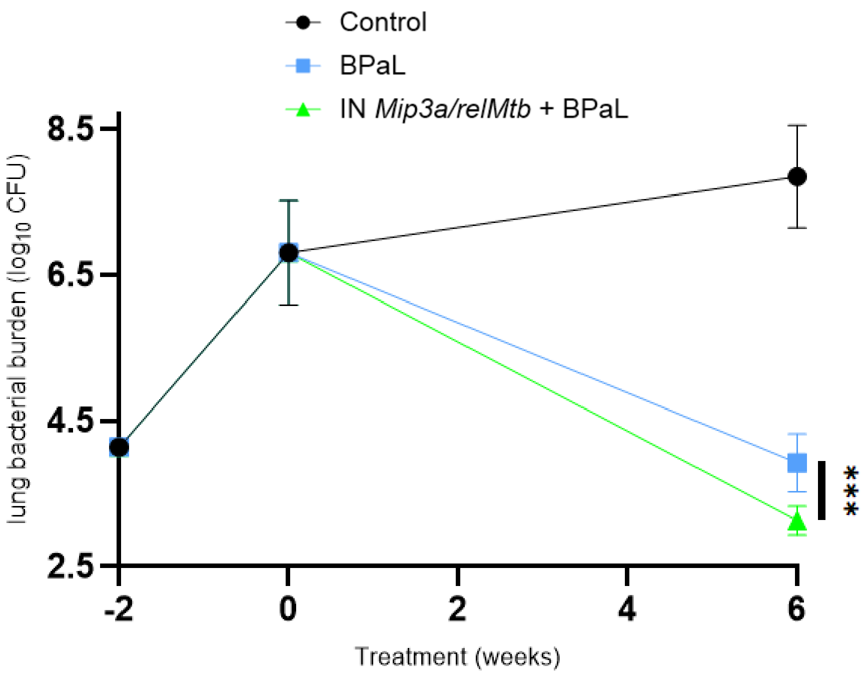

Supplementary Figure 9. IN *Mip3a/relMtb* DNA fusion vaccine enhances the activity of the potent DR-TB regimen BPaL in immunocompletent C57BL6 mice. Timeline of mean lung mycobacterial burden at -2,0 and 6 weeks after treatment initiation; n=10-15/group; one-way analysis of variance followed by Tukey's multiple comparisons tests was used; DR-TB: Drug Resistant TB; Mtb, *Mycobacterium tuberculosis*; IN: intranasal; CFU, colony-forming units; BPaL, Bedaquiline-Pretomanid-Linezolid; P=0.0002

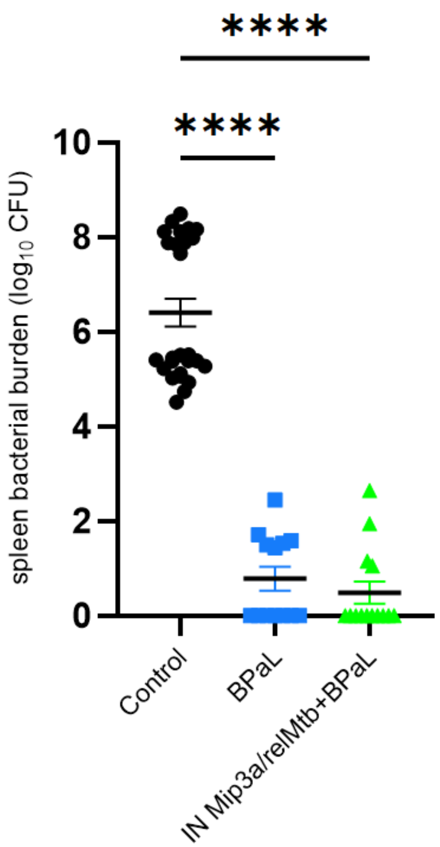

Supplementary Figure 10. Spleen mycobacterial burden do not statistically differ in immunocompetent mice treated with BPaL alone vs. IN *Mip3a/relMtb* fusion vaccine + BPaL. N=10-15/group; one-way analysis of variance followed by Tukey's multiple comparisons tests was used; IN, intranasal; CFU, colony-forming units; BPaL, Bedaquiline-Pretomanid-Linezolid; ****P<0.0001.

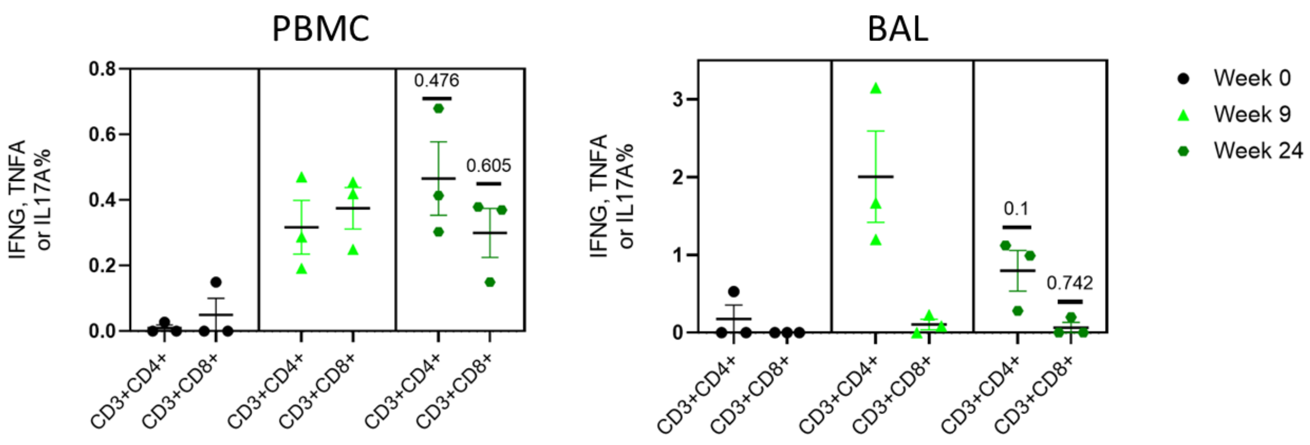

Supplementary Figure 11. IN *Mip3a/relMtb* DNA fusion vaccine induces sustained TB protective immune responses in nonhuman primates by 24 weeks after primary vaccination. Pre-vaccination**,** 9-week and 24-week rel_{Mtb}-specific CD4+ and CD8+T-cell responses (IFNG, TNFA, IL17A) in **(A)** PBMC and **(B)** BAL did not significantly differ over time (comparison between 9 and 24 weeks after primary vaccination). T-cell responses are expressed in percentages; n=3/group; paired t-test was used; IN intranasal; PBMC, peripheral blood mononuclear cells; BAL, bronchoalveolar lavage; prevax, prevaccination.

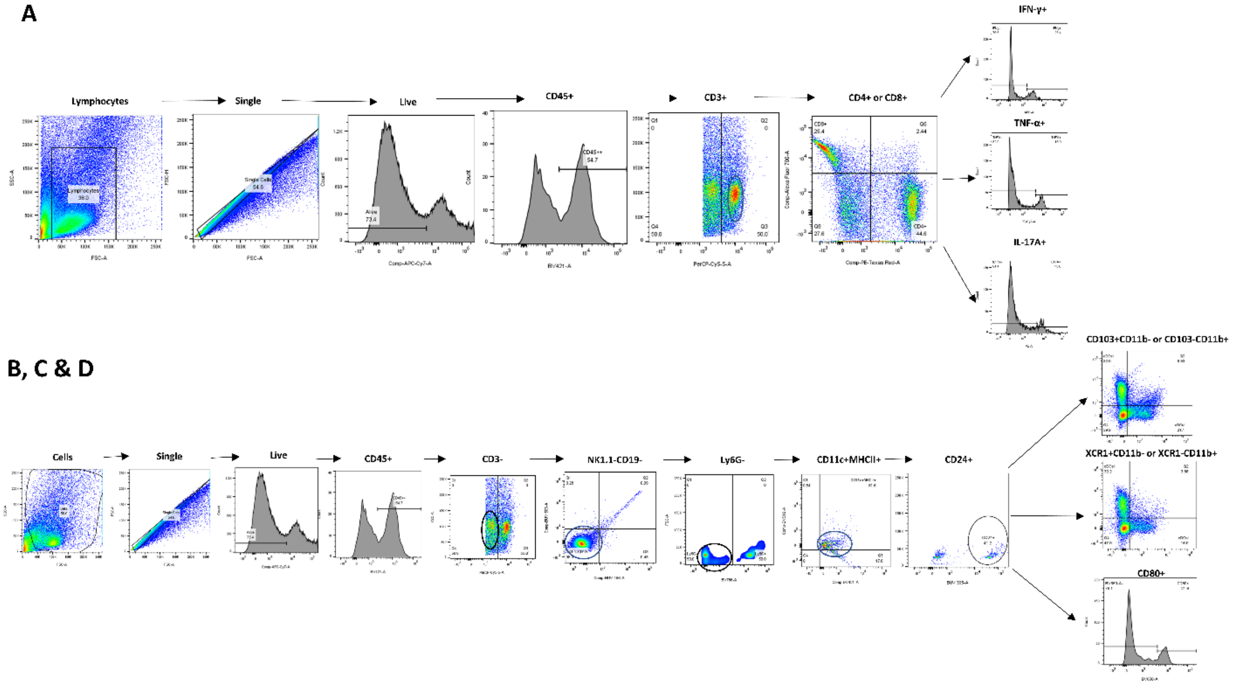

Supplementary Figure 12. Two-dimensional gating strategy for mouse flow cytometric identification of (A) T-cell-producing cytokines, (B) DCs, (C) activated DCs, and (D) lymphoid or non-lymphoid tissue cDC1 and cDC2. We excluded doublets and debris and gated on single live CD45+ cells. **(A)** We identified T cells (CD45+CD3+), CD4+ T cells, CD8+ T cells, and T-cell producing cytokines (CD45+CD3+CD4+IFNG+, TNFA+, or IL17A+ and CD45+CD3+CD8+ IFNG+, TNFA+, or IL17A+); **(B)** classical DCs (CD45+/CD3-CD19-NK1.1-Ly6G-/MHCII+CD11c+/CD24+) **(C)** activated DCs (CD45+/CD3-CD19-NK1.1-Ly6G-/MHCII+CD11c+/CD24+/CD80+) and **(D)** non-lymphoid tissue (lungs or BAL) cDC I (CD45+/CD3-CD19-NK1.1-Ly6G/-MHCII+CD11c+/CD24+/CD103+CD11b-) and cDCII (CD45+/CD3-CD19-NK1.1-Ly6G-/MHCII+CD11c+/CD24+/CD103-CD11b+), and lymphoid tissue (spleen, LNs or PBMCs) cDC I (CD45+/CD3-CD19-NK1.1-Ly6G-/MHCII+CD11c+/CD24+/XCR1+CD11b-) and cDC II (CD45+/CD3-CD19-NK1.1-Ly6G-/MHCII+CD11c+/CD24+/XCR1-CD11b+); DCs, total classical dendritic cells; cDCs I, classical dendritic cells type I; cDCs II, classical dendritic cell type II; PBMC, peripheral blood mononuclear cells; BAL, bronchoalveolar lavage; LNs, mediastinal lymph nodes.

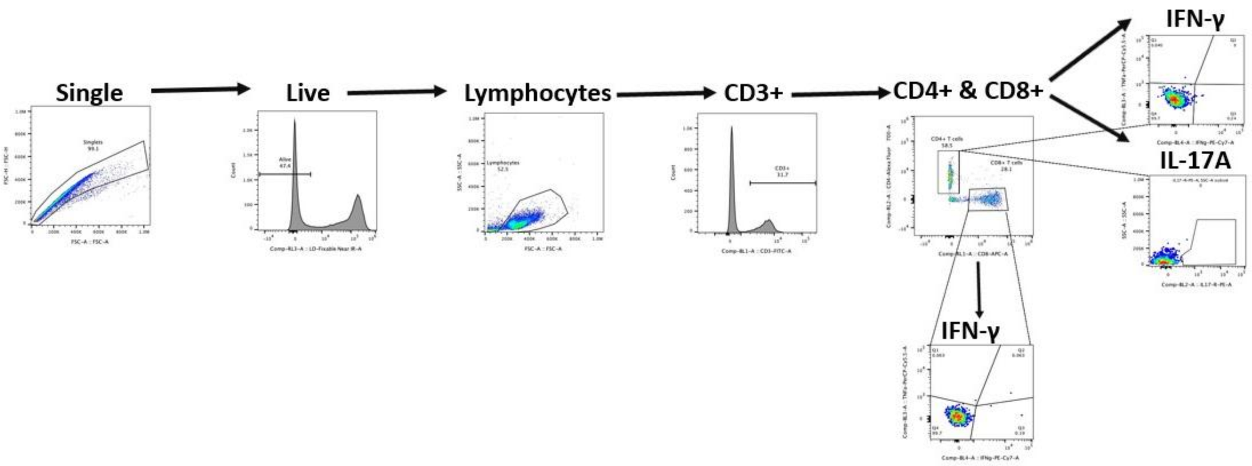

Supplementary Figure 13. Two-dimensional gating strategy for nonhuman primate flow cytometric identification of T-cell-producing cytokines. We excluded doublets and debris and gated on single live cells. We identified T cells (CD3+), CD4+ T cells, CD8+ T cells and T-cell producing cytokines (CD3+CD4+IFNG+, TNFA+, or IL17A+ and CD3+CD8+IFNG+, TNFA+, or IL17A+).
